# 7.10 MAG. A Novel Host Monoacylglyceride for *In Meso* (Lipid Cubic Phase) Crystallization of Membrane Proteins

**DOI:** 10.1021/acs.cgd.4c00087

**Published:** 2024-03-25

**Authors:** Pawel Krawinski, Luke Smithers, Leendert van Dalsen, Coilin Boland, Nikita Ostrovitsa, Javier Pérez, Martin Caffrey

**Affiliations:** † Membrane Structural and Functional Biology Group, School of Medicine and School of Biochemistry and Immunology, Trinity College Dublin, Dublin D02 R590, Ireland; ‡ School of Chemistry, Trinity College Dublin, Dublin D02 R590, Ireland; § SWING Beamline, Synchrotron Soleil, Saint-Aubin 91190, France

## Abstract

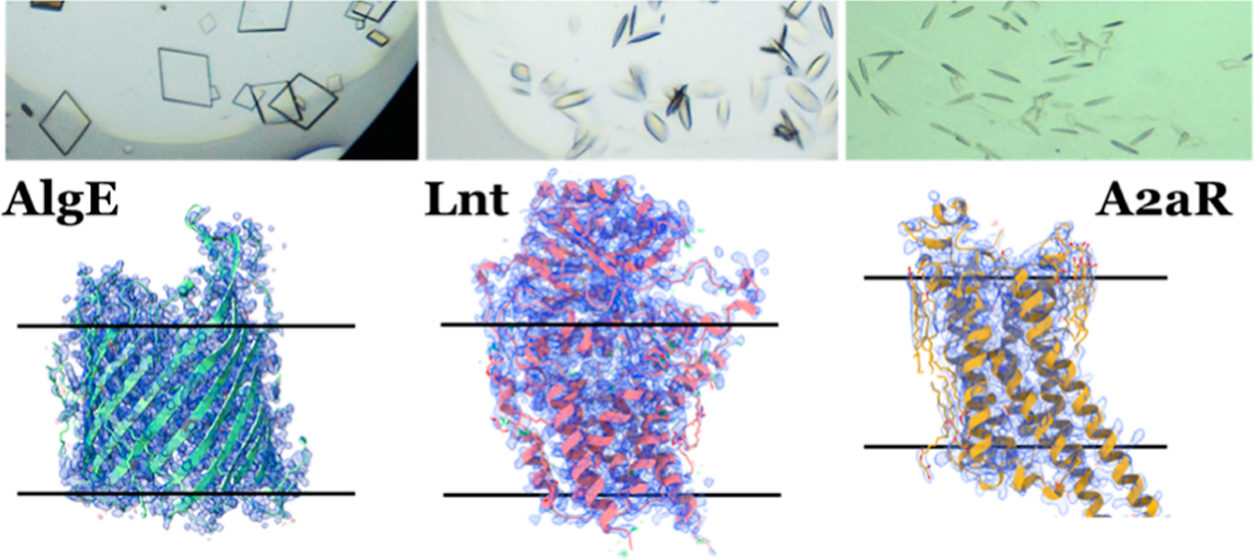

A novel monoacylglycerol, 7.10 MAG, has been produced for use in the *in meso* (lipid cubic phase) crystallization of membrane proteins and complexes. 7.10 MAG differs from monoolein, the most extensively used lipid for *in meso* crystallization, in that it is shorter in chain length by one methylene and its cis olefinic bond is two carbons closer to the glycerol headgroup. These changes in structure alter the phase behavior of the hydrated lipid and the microstructure of the corresponding mesophases formed. Temperature–composition phase diagrams for 7.10 MAG have been constructed using small- and wide-angle X-ray scattering over a range of temperatures and hydration levels that span those used for crystallization. The phase diagrams include lamellar crystalline, fluid isotropic, lamellar liquid-crystalline, cubic-*Ia*3*d,* and cubic-*Pn*3*m* phases, as observed with monoolein. Conspicuous by its absence is the inverted hexagonal phase which is rationalized on the basis of 7.10 MAG’s chemical constitution. The cubic phase prepared with the new lipid facilitates the growth of crystals that were used to generate high-resolution structures of intramembrane β-barrel and α-helical proteins. Compatibility of fully hydrated 7.10 MAG with cholesterol and phosphatidylcholine means that these two lipids can be used as additives to optimize crystallogenesis in screening trials with 7.10 MAG as the host lipid.

## Abstract 2

A new monoacylglycerol, 7.10 MAG, for crystallizing membrane proteins by the *in meso* method is introduced. The cubic phase formed by 7.10 MAG supports the generation of crystals of three membrane proteins, which have been used for high-resolution structure determination. With 7.10 MAG in the *in meso* crystallization toolkit, more effective lipid-screening and optimizations are in prospect.

## 1 Introduction

It is well established that protein structure dictates function.^1^ Although perhaps not immediately obvious, the structure–function principle applies equally well to lipids. Many lipids when hydrated form liquid crystals, also known as mesophases. Mesophases of biological interest include the lamellar (L_α_), hexagonal (H_I_ and H_II_), and cubic phases (cubic-*Pn*3*m*, cubic-*Ia*3*d*, and cubic-*Im*3*m*).^2,3^ Individual mesophases are characterized by distinct microstructural features that include lipid and aqueous layer thicknesses, polar and apolar continuities, and curvature. The mesophase adopted by a lipid depends on its chemical identity or structure, on environmental factors such as temperature and pressure, and on the extent (hydration) and composition of the aqueous component. Mesophase behavior is conveniently and concisely communicated in the form of temperature–composition (*T*–*C*) phase diagrams.^4,5^ Small- and wide-angle X-ray scattering (SAXS and WAXS) are the preferred methods for identifying and structurally characterizing lipid mesophases.^2,3,6,7^

The mesophase properties of lipids engender them with uses in biological systems that include creating life-defining membranes that can accommodate integral and peripheral proteins and that can undergo topological transformations such as fusion and fission.^8−10^ Lipids find applications in processed foods, personal care products, and pharmaceuticals.^4,5,11^ Indeed, most of the billions of COVID-19 vaccine doses administered worldwide in the recent past included a lipid formulation to facilitate drug delivery and efficacy.^12^ All of these applications rely on an appropriate choice of lipid chemical structure to match the particular functional use of that lipid. This is another way of stating that lipid structure, conspiring with environmental and compositional factors, dictates the lipid’s functional properties. It is apparent therefore that understanding the relationship between the chemical structure of a lipid and its functional properties not only is of fundamental physiological and physicochemical interest but also can be exploited practically for the benefit of mankind.

Monoacylglycerides (MAGs), along with diacyl- and triacylglycerides, are a type of lipid of the neutral lipid class.^13^ MAGs are breakdown products of fat digestion.^14^ They find uses as food additives, in part, because of their desirable organoleptic and humectant properties. When hydrated, MAGs can stabilize in a range of different mesophase types in a way that depends on water content and temperature.^2,6,11,15,16^ Respectively, these are called lyotropic and thermotropic phases. Monoolein (9.9 MAG in the *N*.*T* MAG nomenclature)^17^ is a MAG containing oleic acid, an 18-carbon fatty acid with a cis double bond between carbon atoms 9 and 10, in ester linkage at the *sn*-1 hydroxy position of glycerol. When fully hydrated at 20 °C, monoolein adopts the cubic-*Pn*3*m* phase.^18,19^ Under these conditions, the cubic phase is approximately equal parts of monoolein and water. The lipid adopts a familiar form, that of a bilayer hydrated on both sides.^2,20,21^ The packing density of the mesophase is extraordinarily high with a surface area of ∼700 m^2^/g.^22,23^ This is achieved as a result of the bilayer and the bathing aqueous channels on either side being continuous, folded, and highly curved. The cubic phase is termed a bicontinuous or tricontinuous mesophase. In terms of topology, the midplane of the membrane in the cubic phase takes the form of an (infinite) periodic minimal surface.^20,21,24^

**Figure 1:**
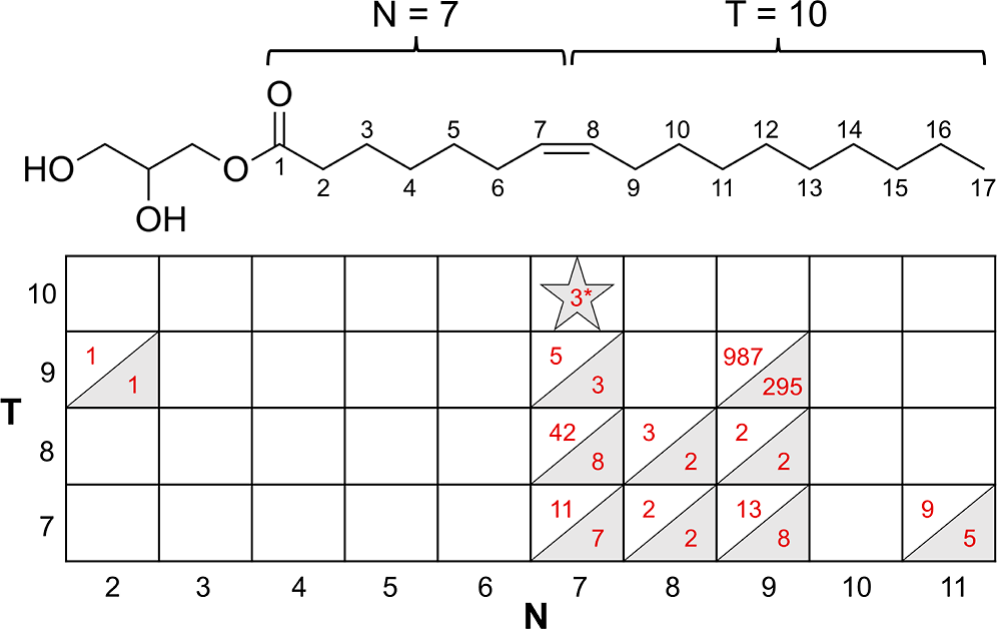
*N*.*T* matrix showing the total and unique number of membrane protein crystal structures solved using different *N*.*T* MAGs. The numbers in the unshaded regions correspond to the total number of protein structures in the Protein Data Bank (RCSB.org)^29^ attributed to a particular *N*.*T* MAG. The numbers in the shaded regions correspond to the unique number of protein structures in the PDB attributed to a particular *N*.*T* MAG. Analysis of the PDB was performed on 9th January 2024. The structure above the matrix explains the *N*.*T*. notation used to describe the host MAG lipids. The data shown for 7.10 MAG were derived from the current study. The asterisk is to alert the reader that while three membrane protein structures (AlgE, Lnt, and A2aR) were solved using crystals grown in 7.10 MAG, a fourth protein, the bacterial diacylglycerol kinase, DgkA,^30^ was crystallized in this MAG and the crystals diffracted to 3.5 Å. However, a structure has not yet been determined for DgkA.

The cubic phase formed by hydrated MAGs has achieved notoriety as a result of its ability to support the growth of membrane protein crystals for use in structure determination by means of X-ray crystallography.^6,25^ In this application, monoolein is the most commonly used so-called host MAG, the lipid that creates the bilayer fabric of the cubic mesophase.^26^ However, monoolein does not work with all membrane proteins. This was recognized early on in the development of the *in meso* crystallization method where MAGs with different acyl chain types were synthesized and screened for one that gave rise to structure-quality crystals.^6,27,28^ Once again, this nicely illustrates the lipid structure–function principle. The logic behind the screening strategy was that different membrane proteins come from different native cellular membranes and compartments, each with its own unique structural and chemical features. Thus, it appeared unlikely that a single host lipid such as monoolein would satisfy the membrane mimetic needs of all protein targets. By creating a suite of MAGs with different chain characteristics, the objective was to provide a more effective means for crystallizing the broadest possible range of membrane protein types. The approach worked as illustrated in Figure 1 which shows the number of different membrane proteins that have yielded structures having been crystallized in cubic phases prepared with different host MAGs.

To exploit the host lipid screen idea more fully, we set about the systematic investigation of the relationship between the MAG identity and mesophase behavior and microstructure. To this end, MAGs, with acyl chains having different lengths and position along the chain of the cis olefin group, were synthesized and purified,^27,28^ and their *T*–*C* phase diagrams were mapped out using SAXS/WAXS. The advantage of SAXS/WAXS is that, in addition to unambiguously identifying phase type, it also enables mesophase microstructure characterization. To date, close to a dozen such phase characterization studies have been reported.^11,18,19,31−41^ A cursory analysis of this data set identifies important and useful relationships. For example, increasing the value of *N* in an *N*.*T* MAG series where *T* is fixed causes H_II_ to dominate at the expense of other liquid-crystalline phases. By contrast, the area occupied by the L_α_ phase in the corresponding phase diagrams drops significantly when the *T* value increases in a MAG series of fixed *N*. Likewise, there are interesting and useful trends in the phase microstructure that reflect changes in chain characteristics. One that was exploited with spectacular results was the effect of reducing chain length while keeping the double bond in the middle of the chain. Thus, in going from 9.9 MAG to 7.7 MAG, the bilayer thickness thinned while the aqueous phase channel diameter of the cubic mesophase increased dramatically.^36^ This enabled the rational use of the latter short-chain MAG for *in meso* crystallization and the structure determination of the β_2_-adrenoreceptor–Gs protein complex that figured in the Nobel Prize in Chemistry in 2012.^42^

While useful information can be gleaned from the existing body of mesophase behavior and related microstructure data, the database itself is sparsely populated. As a result, extrapolations and interpolations to predict the mesophase behavior of novel MAGs based on these data are of somewhat limited utility.^35^ Accordingly, a more complete database is needed to provide for the enhanced reliability of rational design. The current study seeks to contribute to this goal by establishing the phase characteristics of a new lipid, 7.10 MAG. Here, we report on the lyotropic and thermotropic mesophase behavior and microstructure of the lipid in the temperature range from −5 to 105 °C and in the hydration range from 0 to 70% (w/w) water. The compatibility of the mesophase with dioleoylphosphatidylcholine (DOPC) and cholesterol, additive lipids that have been used to optimize crystal growth in *in meso* crystallization trials,^43,44^ is reported. Two distinctly different membrane proteins, one a β-barrel and the other a mix of intramembrane α-helices and an extramembranal αββα sandwich, as well as the α-helical adenosine A_2a_ G protein-coupled receptor (A2aR) were crystallized in 7.10 MAG by the *in meso* method, yielding high-resolution structures in each case. These findings attest to the utility of this lipid for future campaigns aimed at the structure determination of novel membrane proteins and complexes.

## 2 Materials and Methods

### 2.1 Materials

2,5-Hexanediol, cholesterol, cholesteryl hemisuccinate (CHS), 4-(2-hydroxyethyl)piperazineethane-1-sulfonic acid (HEPES), tris(hydroxymethyl)aminomethane (Tris), ethylenediaminetetraacetic acid (EDTA), phenylmethylsulfonyl fluoride (PMSF), polyethylene glycol 400 (PEG400), 2-methyl-2,4-pentanediol (MPD), 2-(*N*-morpholino)ethanesulfonic acid (MES), ammonium sulfate, sodium thiocyanate, potassium thiocyanate, lithium sulfate, sodium citrate, 50 and 100 kDa MWCO Amicon Ultra 15 concentrators, *n*-octyl tetraoxyethylene (C8E4), urea at >99% purity, and ZM241385 were purchased from Sigma-Aldrich. Glycerol, sodium chloride (NaCl), imidazole, kanamycin, Luria broth powder, and isopropyl β-d-1-thiogalactopyranoside (IPTG) were purchased from Thermo Fisher Scientific. Nickel nitrilotriacetic acid (Ni-NTA) Superflow resin was purchased from Qiagen. The HiLoad 16/60 Superdex 200 column and ÄKTA fast protein liquid chromatography system were obtained from GE Healthcare. The detergents 2,2-didecylpropane-1,3-bis-β-d-maltopyranoside (LMNG), *n*-dodecyl-β-D-maltoside (DDM), and *n*-decyl-β-d-maltopyranoside (DM) were purchased from Anatrace. Water (resistivity of 18.2 MΩ·cm) was purified by using a Milli-Q Water Purification System from Millipore. Gastight syringes were obtained from the Hamilton Company. The Xantus liquid and mesophase handling robot was obtained from SIAS. Double-sided 140 μm thick spacers were purchased from Saunders Corp. Glass crystallization plates and cover slides were obtained from Marienfeld. Harvesting loops were purchased from Mitegen.

### 2.2 7.10 MAG Synthesis and Purification

A detailed description of the methods used for synthesizing and purifying 7.10 MAG is included in Supporting Information (Figures S1–S4).

### 2.3 Sample Preparation

Samples for phase diagram construction were prepared over a range of water contents from 0 to 70% (w/w) using a coupled syringe-mixing device as described.^45,46^ Samples for cholesterol and DOPC compatibility studies were prepared at 60% (w/w) water over a range of cholesterol contents from 0 to 30 mol % and DOPC from 0 to 30 mol % in 7.10 MAG as described.^47^ Homogeneous samples were transferred to 1 mm diameter thin-walled glass X-ray capillaries (HR6-104. Hampton Research Corp., Aliso Viejo, CA) using a custom-made 100 mm long, 22-gauge, end point style 3 needle (Hamilton Company, Reno, NV, Part no. 7804-01) and centrifuged (Eppendorf centrifuge 5810 R, Eppendorf, Hamburg, Germany) at 40 °C and 4000 rpm to the bottom of the capillary. Loaded capillaries were flame- and epoxy-sealed (Araldite Rapid Epoxy Adhesive, Pearse Street Hardware Ltd., Dublin) and stored at 20 °C prior to data collection. A movie describing sample preparation and capillary loading and sealing is available in Supporting Information (Movie S1).

### 2.4 Small- and Wide-Angle X-ray Scattering

Diffraction measurements were made at the Soleil Synchrotron SWING beamline by using a 15 keV (0.827 Å) beam measuring 350 μm wide (fwhm) and 25 μm high at the sample. The incident flux was (3–5) × 10^12^ photons/s. For each diffraction measurement at a fixed location in the sample capillary, five sequential 200 ms images were recorded by using an Eiger X4M detector with a pixel size of 75 μm at a sample-to-detector distance of 519 mm. No evidence of radiation damage was observed across the image stack. Accordingly, five images were averaged and used for subsequent data analysis.

Samples were housed in the SWING beamline Static Capillary Holder (SCH) (Figure S5) with temperature regulated in the range from −10 to 110 °C. The sample temperature was adjusted in increments of 1 to 10 °C. Diffraction measurements were made between 10 and 30 min after the sample temperature had stabilized at its target value. The SCH was placed in the beam, with the long axis of the capillaries oriented vertically. Between measurements, capillaries were translated by 30 μm along the capillary axis to minimize radiation damage. Radial averaging of the recorded powder diffraction patterns through 10 to 360° was used to generate intensity versus scattering angle (I–2θ) plots. The diffraction data were analyzed for phase identification and lattice parameter determination as described.^7,46^

### 2.5 Protein Production and Crystallization

Lnt (*Escherichia coli*, Uniprot ID P23930), AlgE (*Pseudomonas aeruginosa*, Uniprot ID P0ABN1), and A2aR (Human, Uniprot ID P29274) proteins were produced recombinantly following established protocols.^48−50^ Briefly, the Lnt wild-type (WT) DNA from *E. coli* (Lnt) was cloned into the pET28a plasmid, and the protein was expressed in C43(DE3) cells. The hexahistidine-tagged LntEco WT protein was solubilized in LMNG and purified using nickel affinity chromatography, followed by size exclusion chromatography.^48^ The Lnt protein was concentrated to 13 mg/mL in **Buffer A** [20 mM sodium citrate, pH 6.0, 200 mM NaCl, 10% (v/v) glycerol, 0.05% (w/v) LMNG]. AlgE WT DNA from *P. aeruginosa* was cloned into the pET28a plasmid, and the protein was expressed in BL21(DE3) RIL cells. The hexahistidine-tagged AlgE protein was solubilized in urea, refolded in DM, and purified using nickel affinity chromatography, followed by size exclusion chromatography which also exchanged the detergent from DM to C8E4.^49^ The pure protein was concentrated to 35 mg/mL in **Buffer B** [20 mM Tris–HCl pH 8.0, 150 mM NaCl, 0.45% (v/v) C8E4]. A2aR with the thermostabilized apocytochrome b_562_ (BRIL) fusion protein was cloned into the pFastBac1 plasmid, and recombinant baculoviruses were produced by the Bac-to-Bac method (Invitrogen). The protein was expressed by infecting *Spodoptera frugiperda* (Sf9) insect cells with the recombinant baculovirus. The decahistidine-tagged A2aR was solubilized in DDM and CHS and purified using cobalt affinity chromatography.^50^ The A2aR protein was concentrated to 60 mg/mL in **Buffer C** [25 mM HEPES pH 7.5, 800 mM NaCl, 220 mM imidazole pH 7.5, 10% (v/v) glycerol, 0.025% (v/v) DDM, 0.005% (v/v) CHS, 25 μM ZM241385].

Mesophase for *in meso* crystallization of Lnt and AlgE was prepared by combining 3 volumes of dry molten 7.10 MAG with 2 volumes of protein solution.^51^ For A2aR, the 7.10 MAG was first doped with 10% (w/w) cholesterol and the mesophase prepared as for Lnt and AlgE. Lnt at 13 mg/mL in Buffer A, AlgE at 35 mg/mL in Buffer B, and A2aR at 60 mg/mL in Buffer C were used for protein-laden mesophase preparation with a coupled syringe-mixing device.^52,53^*In meso* crystallogenesis was performed with a Sias Xantus robot, 96-well glass sandwich plates incorporating 140 μm thick double-stick spacers, and with 50 nL protein-laden mesophase and 800 nL precipitant solution per well.^52,54^ The crystallization screen used for Lnt was 100 mM MES pH 6.0, 8% (v/v) MPD, and 50–400 mM sodium or potassium thiocyanate in 48 steps. For AlgE, screening was done with precipitant solutions containing 34–41% (v/v) PEG400, 100 mM LiSO_4_, and 100 mM Na-citrate at pH 5.6 and 6.0 and 34–41% (v/v) PEG400, 25–300 mM (NH_4_)_2_SO_4_, and 100 mM Na-citrate at pH 5.6 and 6.0. For A2aR, the precipitant screen used contained 30–35.5% (v/v) PEG400, 50 mM sodium thiocyanate, 100 mM sodium citrate at pH 5, 0.2%(v/v) 2,5-hexanediol, and 25 μM ZM241385. Plates were incubated and imaged in a Formulatrix RI1500 imager at 20 °C. Crystals of Lnt, AlgE, and A2aR were harvested with MiTeGen loops, 21, 30, and 14 days post-setup, respectively, and were snap-cooled in liquid nitrogen without added cryogen.^55^

### 2.6 Macromolecular X-ray Crystallography

Diffraction data were collected at the Diamond Light Source (DLS) I24 beamline and the Swiss Light Source (SLS) PXI beamline. Data sets were indexed using the X-ray Detector Software^56^ and scaled and merged using StarAniso.^57^ In the case of AlgE and A2aR, crystal packing was identical to that observed in the previously solved structures (PDB ID, 4AFK for AlgE, 4EIY for A2aR) obtained with crystals grown in 9.9 MAG. The set of reflection flags for free R used with the 4AFK and 4EIY search models were reused and extended to the higher resolution of 1.45 Å for AlgE to ensure the free R reflections remained independent of the final model. As the symmetry observed with Lnt crystals grown in 7.10 MAG was different from that found in the previously solved search model structure (5N6H), a new set of free R reflections was generated. The maximum likelihood molecular replacement was carried out using Phaser MR (within CCP4)^58,59^ with the relevant search model (4AFK for AlgE,^60^ 5N6H for Lnt,^48^ and 4EIY for A2aR).^50^ Refinement was performed using Phenix Refine within the Phenix suite of programs^61^ with model building in COOT.^62^ Restraint and coordinate files for 7.10 MAG were generated using ACEDRG in CCP4.^59,63^ Data collection and refinement statistics are listed in Table 1.

**Table 1:** Data Collection and Refinement Statistics for AlgE, Lnt, and A2aR Crystallized in 7.10 MAG^a^

	AlgE (PDB ID, 8RQP)	Lnt (PDB ID, 8RQR)	A2aR (PDB ID, 8RQQ)
Data Collection Statistics			
space group	*C*2	*P*2_1_22_1_	*C*222_1_
*a*, *b*, *c* (Å)	57.4, 74.5, 115.9	48.7, 76.5, 157.1	39.7, 180.4, 141.5
α, β, γ (deg)	90, 102.5, 90	90, 90, 90	90, 90, 90
beamline	SLS-PXI	DLS-I24	DLS-I24
wavelength (Å)	0.98	0.97	1
resolution (Å)	39.4–1.45 (1.50–1.45)	46.50–2.19 (2.27–2.19)	42.98–2.37 (2.46–2.37)
no. of rflns (total/unique)	417,256/61,350	197,541/30,881	427,494/16,601
*Rpim*	0.10 (0.80)	0.10 (0.85)	0.13 (0.6)
I/σI	7.2 (1.6)	7.7 (1.0)	6.8 (1.4)
completeness (%)	90.7 (48.9)	99.1 (94.6)	89.5 (42)
multiplicity	6.8 (7.4)	6.4 (6.4)	20.1 (20)
CC1/2	0.99 (0.27)	0.99 (0.31)	0.99 (0.46)
Refinement Statistics			
resolution (Å)	39.4–1.45 (1.50–1.45)	46.50–2.19 (2.27–2.19)	42.98–2.37 (2.46–2.37)
no. of rflns	61,250 (310)	30,739 (2879)	16,564 (165)
Rwork/Rfree	0.19/0.22	0.20/0.24	0.22/0.27
rms deviations			
bond lengths (Å)	0.013	0.003	0.004
bond angles (deg)	1.19	0.72	0.65
no. of non-hydrogen atoms			
protein	3697	4032	3036
ligands	759	551	833
waters	234	126	36
Ramachandran plot			
favored (%)	96.69	97.04	98.19
allowed (%)	3.31	2.76	1.81
outliers (%)	0	0.2	0
*B* factors			
average	23.46	40.69	56.4
protein	21.63	39.46	56.1
ligands	42.28	65.13	60.7
waters	27.97	37.91	40.5

a Values in parentheses are for the highest resolution shell.

## 3 Results

### 3.1 Phase Characterization

#### 3.1.1 Operational Phase Diagram and Phase Microstructure

**Figure 2:**
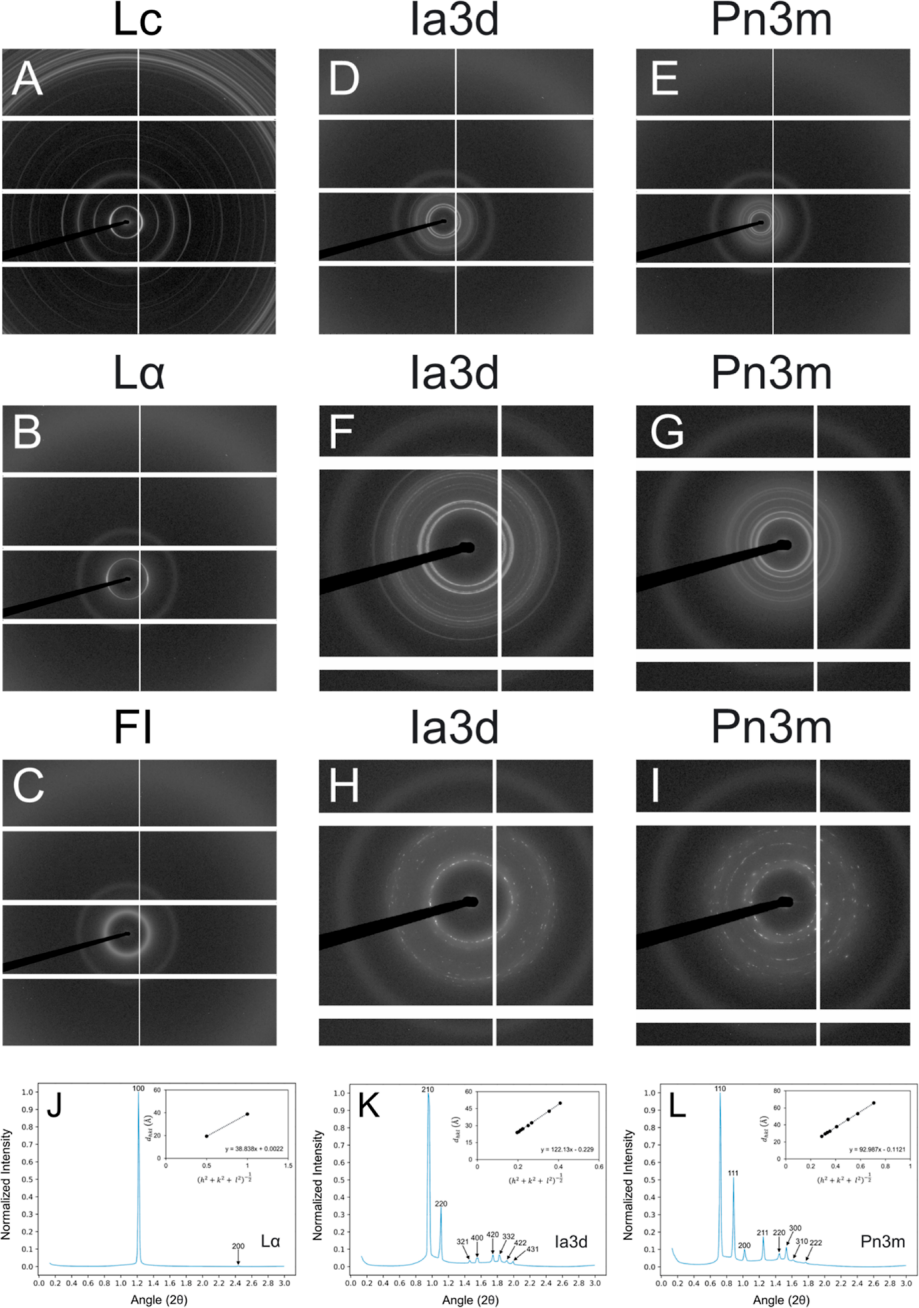
Diffraction patterns of the different solid, liquid, and liquid-crystalline phases observed for 7.10 MAG at different hydration levels and different temperatures. (A–I). 2D diffraction patterns. (A) 7.10 MAG at 15% (w/w) water and 5 °C in the Lc phase. WAXS and SAXS. (B) 7.10 MAG at 10% (w/w) water and 25 °C in the L_α_ phase. WAXS and SAXS. (C) 7.10 MAG at 5% (w/w) water and 95 °C in the FI phase. WAXS and SAXS. (D) 7.10 at 35% (w/w) water and 25 °C in the *Ia*3*d* phase. WAXS and SAXS. (E) 7.10 MAG at 60% (w/w) water and 25 °C in the cubic-*Pn3m* phase. WAXS and SAXS. (F) 7.10 MAG at 35% (w/w) water and 25 °C in the cubic-*Ia3d* phase. SAXS. (G). 7.10 MAG at 60% (w/w) water and 25 °C in the cubic-*Pn3m* phase. SAXS. (H). 7.10 MAG at 30% (w/w) water and 25 °C in the cubic-*Ia*3*d* phase. SAXS, spotty pattern. (I). 7.10 MAG at 45% (w/w) water and 25 °C in the cubic-*Pn*3*m* phase. SAXS, spotty pattern. (J–L) I–2θ plots along with phase identification, indexing, and lattice parameter determination as insets. (J) 7.10 MAG at 15% (w/w) water and 25 °C in the L_α_ phase. (K) 7.10 MAG at 30% (w/w) water and 27 °C in the *Ia*3*d* phase. (L). 7.10 MAG at 40% (w/w) water and 25 °C in the *Pn*3*m* phase. Key: *d*_(*hkl*)_, experimentally determined *d*-spacing value; *h*, *k*, *l*, Miller indices of Bragg reflections. The slope of the line of best fit is the lattice parameter of the phase with the corresponding values of 38.8, 122.1, and 93.0 Å for the L_α_, cubic-*Ia*3*d*, and cubic-*Pn*3*m* phases, respectively.

As noted, SAXS and WAXS are reliable methods for unambiguously characterizing solid, liquid, and mesophase identity and microstructure. Exemplar diffraction patterns for all phases encountered in this study are shown in Figure 2. These include the Lc, L_α_, cubic-*Pn*3*m*, cubic-*Ia*3*d*, and fluid isotropic (FI) phases. Most patterns are well “powdered” showing approximately uniform intensity around the diffraction ring. Others are spotty, indicating a tendency for the mesophase to form crystallites or microdomains, as illustrated in Panels 2H and 2I in Figure 2.

The first phase characterization study performed on 7.10 MAG was designed to generate what we refer to as an operational *T*–*C* phase diagram. It is so named to reflect the manner in which a protein-laden mesophase is prepared and used in crystallization trials. This involves homogenizing lipid and protein solution at room temperature (RT, typically 20–22 °C) to form the cubic phase which is then incubated at a fixed temperature, usually either 20 or 4 °C, during which crystals grow. Accordingly, two sets of samples for use in generating the operational phase diagram were prepared at different hydration levels ranging from 0 to 70% (w/w) water at RT. SAXS/WAXS data were collected on one set in the heating direction from RT (25 °C at the synchrotron, where diffraction measurements were made) to above 100 °C until the FI phase was accessed. The second set of samples was used for data collection in the cooling direction from RT to −5 °C. Under these conditions, phase undercooling is commonly encountered.^18,19,34,38,39^ Thus, a phase that is thermodynamically unstable at low temperatures continues to exist in an undercooled state that can persist for extensive periods. This undercooling feature of the cubic phase has been exploited in crystallization trials performed with monoolein at 4 °C. For reference, under the so-called equilibrium conditions, the cubic phase of fully hydrated monoolein transitions to a solid Lc phase at ∼17 °C.^38^

**Figure 3:**
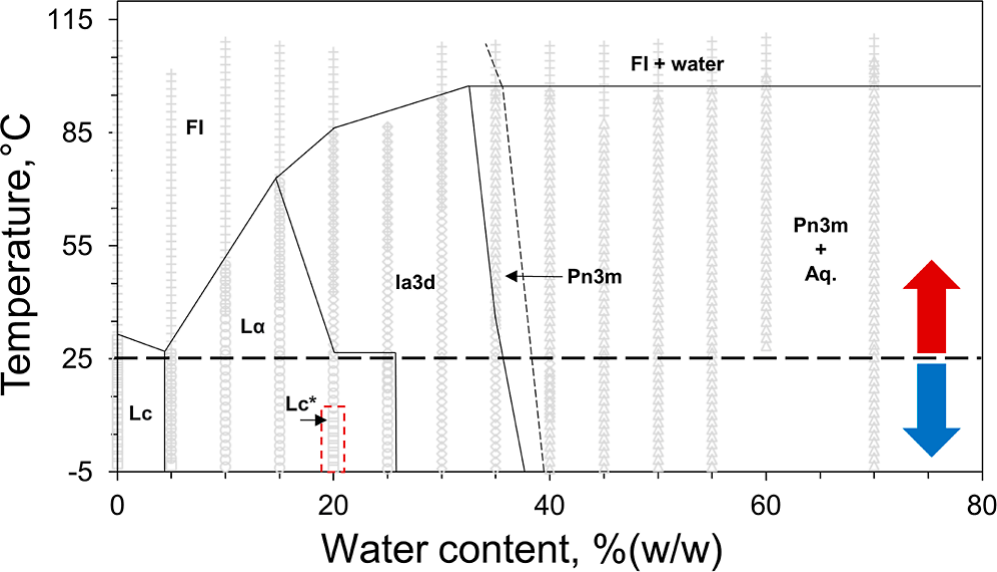
Operational phase diagram for the 7.10 MAG/water system. Phases were identified by SAXS and WAXS measurements recorded in the cooling (blue arrow) and heating (red arrow) directions from ∼25 °C (dark dashed horizontal line). The identity of each of the phases is as follows: (□) Lc, (○) L_α_, (◊) cubic-*Ia*3*d*, (△) cubic-*Pn*3*m*, and (+) FI. Temperature readings are reliable to ±1 °C. The maximum hydration boundaries for the cubic-*Pn*3*m* and FI phases are estimated and are shown as dashed lines. Spontaneous transitioning from an undercooled L_α_ phase to a more stable Lc phase likely occurred in the 20% (w/w) water sample in the 10 to −5 °C range (dashed red lines). Phase boundaries are approximate and have been drawn to guide the eye.

The operational *T*–*C* phase behavior of 7.10 MAG is shown in Figure 3. In many ways, it resembles the behavior observed with related *N*.*T* MAGs in that the familiar Lc, L_α_, cubic-*Ia*3*d*, cubic-*Pn*3*m*, and FI phases are seen in the expected locations in the *T*–*C* phase diagram. In the heating part of the diagram above 25 °C, the liquid crystal and fluid phases dominate. The dry sample undergoes melting from the solid, Lc, to the liquid, FI, phase which is complete at 32 °C. The low-temperature boundary of the FI phase rises from a low temperature of ∼27 °C at 5% (w/w) water to a high temperature of ∼96 °C at and above ∼35% (w/w) water. The cubic-*Pn*3*m* phase prevails in the region of full hydration which extends from ∼40% (w/w) water at 25 °C to ∼35% (w/w) water at 96 °C. Reducing the sample water content along the 25 °C isotherm sequentially gives rise to the cubic-*Ia*3*d* phase, followed by the L_α_ and Lc phases.

**Figure 4:**
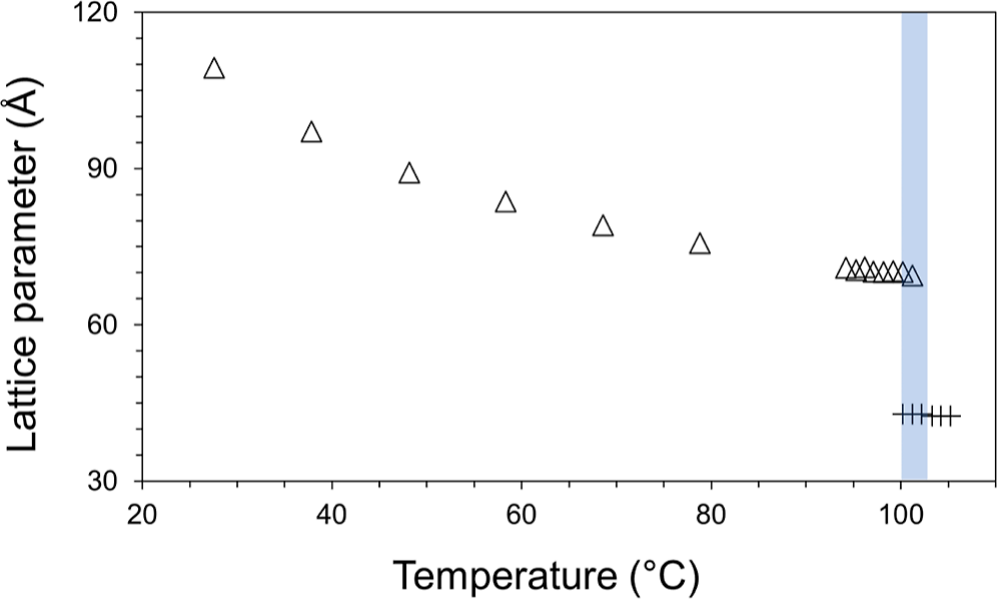
Evidence that fully hydrated 7.10 MAG does not form the H_II_ phase and that it transitions directly from the cubic-*Pn*3*m* to the FI phase with increasing temperature. Under these conditions, the transition begins at ∼100 °C and is complete by ∼103 °C (shaded region) at an average heating rate of 6 °C/h. Data shown are the structure parameters of the cubic-*Pn*3*m* (△) and FI (+) phases. The sample used in this measurement was prepared with 60% (w/w) water.

Conspicuous by its absence in the *T*–*C* phase diagram of the 7.10 MAG is the H_ll_ phase. In related *N*.*T* MAG phase diagrams, this inverted phase appears at high temperatures between the other liquid-crystalline phases and the FI phase.^18,34,40^ However, the temperature range in which the H_II_ phase exists can be small. Accordingly, there is the possibility that in a study such as the current one, where temperature adjustments are made in increments of 2 °C with incubation times of 20 min at each temperature, the H_II_ phase could be missed. To mitigate this risk, a careful study was conducted of phase behavior in the vicinity of the cubic-*Pn*3*m*-to-FI phase boundary in a fully hydrated 7.10 MAG sample. Measurements were made every 1 °C in the temperature range from 94 to 105 °C with an incubation time of 10 min at each temperature. Under these more probing measurement conditions, only the cubic-*Pn*3*m* and FI phases were observed; the H_II_ phase was not in evidence (Figure 4). This finding is consistent with predictions, as discussed below.

**Figure 5:**
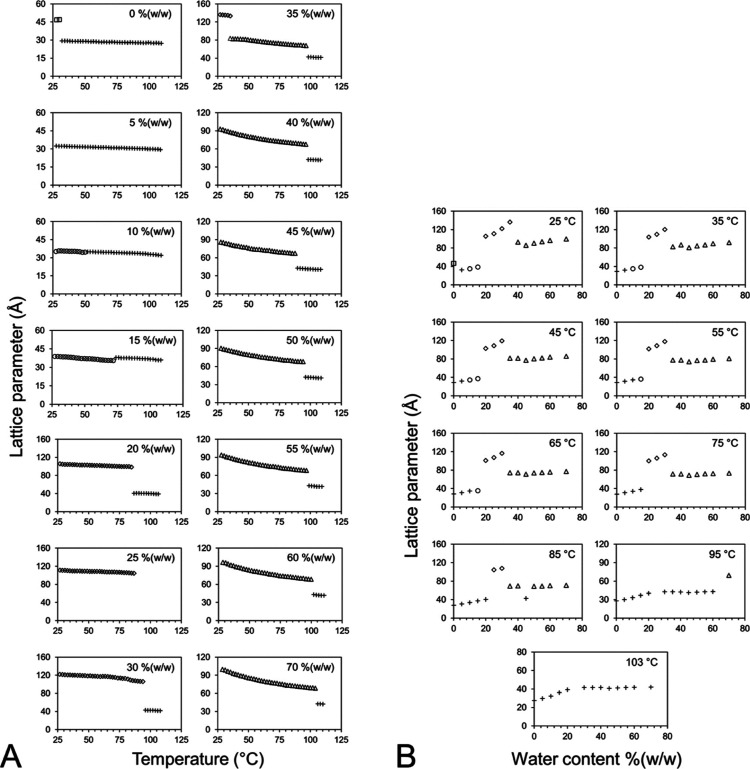
Dependence of the structure parameters of the phases formed by the 7.10 MAG/water system on temperature (A) and water content (B) recorded in the heating direction from 25 to 105 °C. Samples were prepared and stored at RT until used in this heating study. The identity of each of the phases is as follows: (□) Lc, (○) L_α_, (◊) cubic-*Ia*3*d*, (△) cubic-*Pn*3*m*, and (+) FI.

One of the advantages of SAXS measurements is that in addition to phase identification, structure parameters for the different phase states can be derived from the diffraction data. These are presented in Figure 5. Figure 5A shows the structure parameters for the different phases recorded in the heating direction in the *T*–*C* phase diagram plotted as a function of temperature. As expected, for all phase states, the structure parameter drops with increasing temperature. For most phases in single-phase regions, the change with temperature is small. This reflects the cis/trans isomerization or disorder in the acyl chains, which increases with temperature, causing the bilayers to reduce in thickness and for the size of the structure parameters to drop. The change in the structure parameter is more dramatic in the cubic-*Pn*3*m* phase under conditions of full hydration. Here, at least two effects are responsible for the decrease. First, there is the cis/trans isomerization of acyl chains, as noted. The second relates to the fact that the cubic phase dehydrates with increasing temperature. This is evidenced by the full hydration boundary of the phase depicted as moving to lower hydration levels with rising temperature (Figure 3).

The hydration dependence of the structure parameters of the different phases expressed by 7.10 MAG in the heating direction across the *T*–*C* phase diagram is shown in Figure 5B. For a single phase, increasing the water content is expected to cause the lattice parameter to rise as the phase swells. This is what was observed in the current study. Imbibition is particularly well developed in the case of the *Ia*3*d* phase. By comparison, the cubic-*Pn*3*m* phase is, within noise, insensitive to the hydration level. This indicates that the mesophase is in equilibrium with excess water over the entire range of water contents examined. It also means that the pure cubic-*Pn*3*m* phase exists over a narrow, yet undetermined, hydration range. It is for this reason that a dashed line is used in Figure 3 to indicate a putative hydration limit for the cubic-*Pn*3*m* phase.

The operational *T*–*C* phase behavior of 7.10 MAG in the cooling direction from 25 to −5 °C is shown in Figure 3. Across the entire hydration range investigated, the phases observed at RT persist down to −5 °C. As will be apparent from the approach-to-equilibrium phase diagram presented in the following section, this finding clearly demonstrates the ability of the liquid-crystalline phases to undercool. Given such behavior, 7.10 MAG should prove useful for crystallization screening trials conducted at 4 °C, as has been done with other *N*.*T* MAGs. However, under these conditions, the cubic phase would exist in a thermodynamically unstable state and could transition to the more stable solid phase at any point. Indeed, we see an example of this in the 20% (w/w) water sample included in this study (Figure 3, dashed boxed area in red). This sample starts out in the L_α_ phase at 25 °C and remains so upon cooling to ∼12 °C. At ∼10 °C, it transitions to the solid Lc phase and remains solid upon continued cooling.

**Figure 6:**
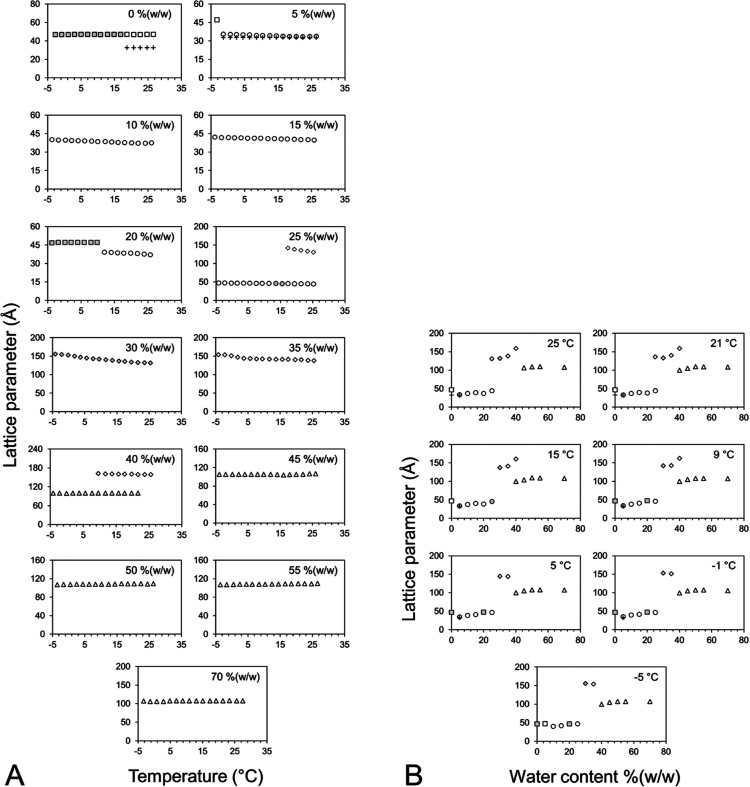
Dependence of the lattice parameters of the phases formed by the 7.10 MAG/water system on temperature (A) and water content (B) recorded in the cooling direction from 25 to −5 °C. Samples were prepared and stored at RT until used in this cooling study. The identity of each of the phases is as follows: (□) Lc, (○) L_α_, (◊) cubic-*Ia*3*d*, (△) cubic-*Pn*3*m*, and (+) FI.

Changes in the lattice parameters of the different phases in the cooling direction as a function of temperature are shown in Figure 6A. As expected, the general behavior is for these parameters to rise with decreasing temperature in single-phase regions. The lattice parameter of the cubic-*Pn*3*m* phase, in contrast, remains constant with temperature in the range studied. This suggests that the boundary for full hydration is relatively insensitive to temperature, as will be discussed. The compositional dependence of lattice parameters of the different phases formed by 7.10 MAG recorded in the cooling direction is shown in Figure 6B. Once again, the expected increase in lattice parameter with increasing water content in single-phase regions is observed.

#### 3.1.2 Approach-to-Equilibrium Phase Behavior and Phase Microstructure

The operational *T*–*C* phase diagram described in the previous section was created in an attempt to mimic the phase behavior expected for protein-laden mesophase handled as it would be in setting up *in meso* crystallization trials. In so doing, phase undercooling is encountered. To avoid undercooling and metastable behavior, data have been collected under what we refer to as “approach-to-equilibrium” conditions. In this case, the samples are initially quenched in liquid nitrogen to set all in the low-temperature Lc phase. Samples are then raised to −5 °C and incubated there for 40 min before being used for SAXS/WAXS measurement in the temperature range from −5 to 65 °C. In this way, the liquid-crystalline phases are accessed in the heating direction from the solid state where undercooling is not an issue.

**Figure 7:**
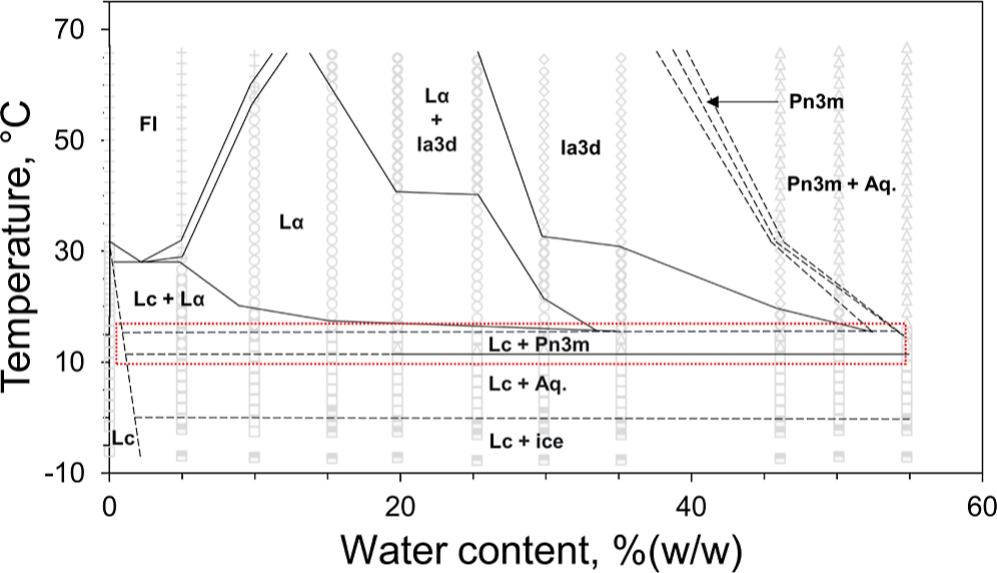
Phase behavior of the 7.10 MAG/water system recorded in the heating direction under approach-to-equilibrium conditions. Phases were identified by SAXS measurements on homogeneous lipid/water samples quenched to liquid nitrogen temperatures and held at −5 °C for 40 min before being heated in steps of 2 °C with incubation times of 20 min at each temperature. Solid lines correspond to phase boundaries as defined by the SAXS and WAXS data. Dashed lines correspond to boundaries that are assumed to exist on the basis of data collected on related systems and on the Gibbs phase rule. No attempt has been made to conform to the phase rule in the boxed region of the diagram (red dotted box) which is expected to have many areas of phase coexistence based on the observations made with the related system. The identity of each of the phases is as follows: (□) Lc, (□) Lc plus ice, (○) L_α_, (◊) cubic-*Ia*3*d*, (Δ) cubic-*Pn*3*m*, and (+) FI. Symbols with shading indicate the presence of trace amounts of an unidentified phase or phases.

**Figure 8:**
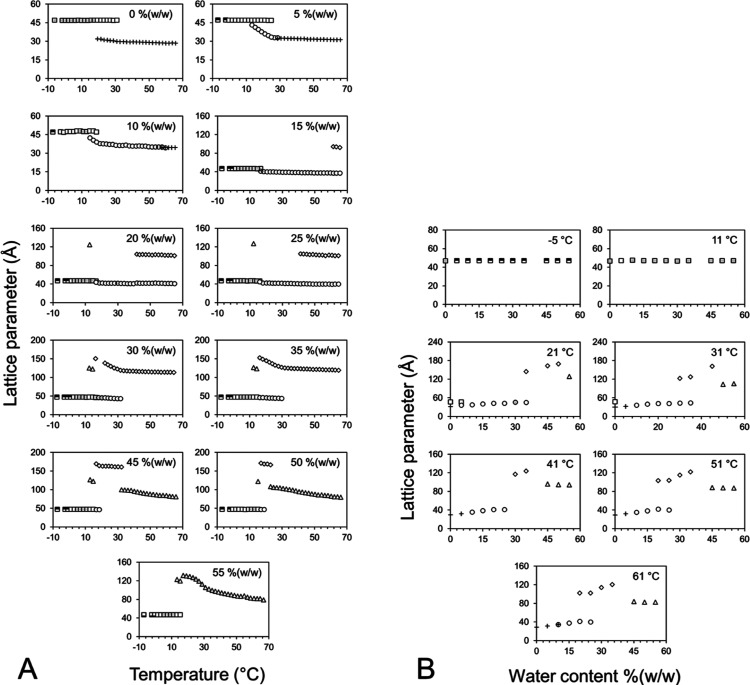
Dependence of the lattice parameters of the phases formed by the 7.10 MAG/water system on temperature and water content under approach-to-equilibrium data collection conditions. Lattice parameters were determined based on SAXS measurements recorded in the heating direction from −5 to 65 °C following sample quenching to liquid nitrogen temperatures and incubation at −5 °C for 40 min. The identity of each of the phases is as follows: (□) Lc, (□) Lc plus ice, (○) L_α_, (◊) cubic-*Ia*3*d*, (△) cubic-*Pn*3*m*, and (+) FI. Symbols with shading indicate the presence of trace amounts of an unidentified phase or phases.

The approach-to-equilibrium phase diagram is shown in Figure 7. The corresponding lattice parameter data as a function of the temperature and hydration level are presented in Figure 8. The phase diagram shows clearly that the solid, low-temperature Lc phase is stable up to ∼15 °C under conditions of full hydration. This means that the persistence of the cubic-*Pn*3*m* phase to −5 °C observed in the cooling direction in the operational phase diagram (Figure 3) represents undercooling. Accordingly, while crystallization trials can be set up with 7.10 MAG at 4 °C, as required for some proteins, the mesophase is in an undercooled state and may transition at any point to the Lc phase, which will terminate the crystallization process. As noted, however, undercooling can persist for extended periods, during which crystals can grow and be harvested successfully.

At and above 20 °C, the approach-to-equilibrium (Figure 3) and operational phase diagrams (Figure 7) are similar. Likewise, the lattice parameter dependence of the different phase states on the temperature and composition follows analogous trends (Figure 8). However, notable differences between the two do exist. One, in particular, refers to the hydration limits recorded for the liquid-crystalline phases. While the accuracy in the positioning of these boundaries is low given that the hydration axis is sampled in increments of just 5% (w/w) water, it is apparent that the boundaries in the “equilibrium” phase diagram are shifted to the right in comparison with those in the operational diagram. This means that the phases have been captured in a more hydrated state under “equilibrium” data collection conditions. One explanation for this is that the rate at which the “equilibrium” data were gathered is too fast such that the nascent liquid-crystalline phases that evolve from the Lc phase upon heating do not have enough time to equilibrate by shedding water and for the corresponding hydration boundaries to move to the left in the phase diagram. By contrast, the operational phase diagram data were collected in the heating direction from 25 °C using samples that had been prepared and equilibrated at RT for weeks in advance of diffraction measurements. Accordingly, the data shown in the heating part of the operational phase diagram likely more closely reflect the equilibrium behavior. While the differences between the two phase diagram types are small, they are worth noting.

### 3.2 Compatibility of the 7.10 MAG Cubic Phase with Cholesterol and DOPC

**Figure 9:**
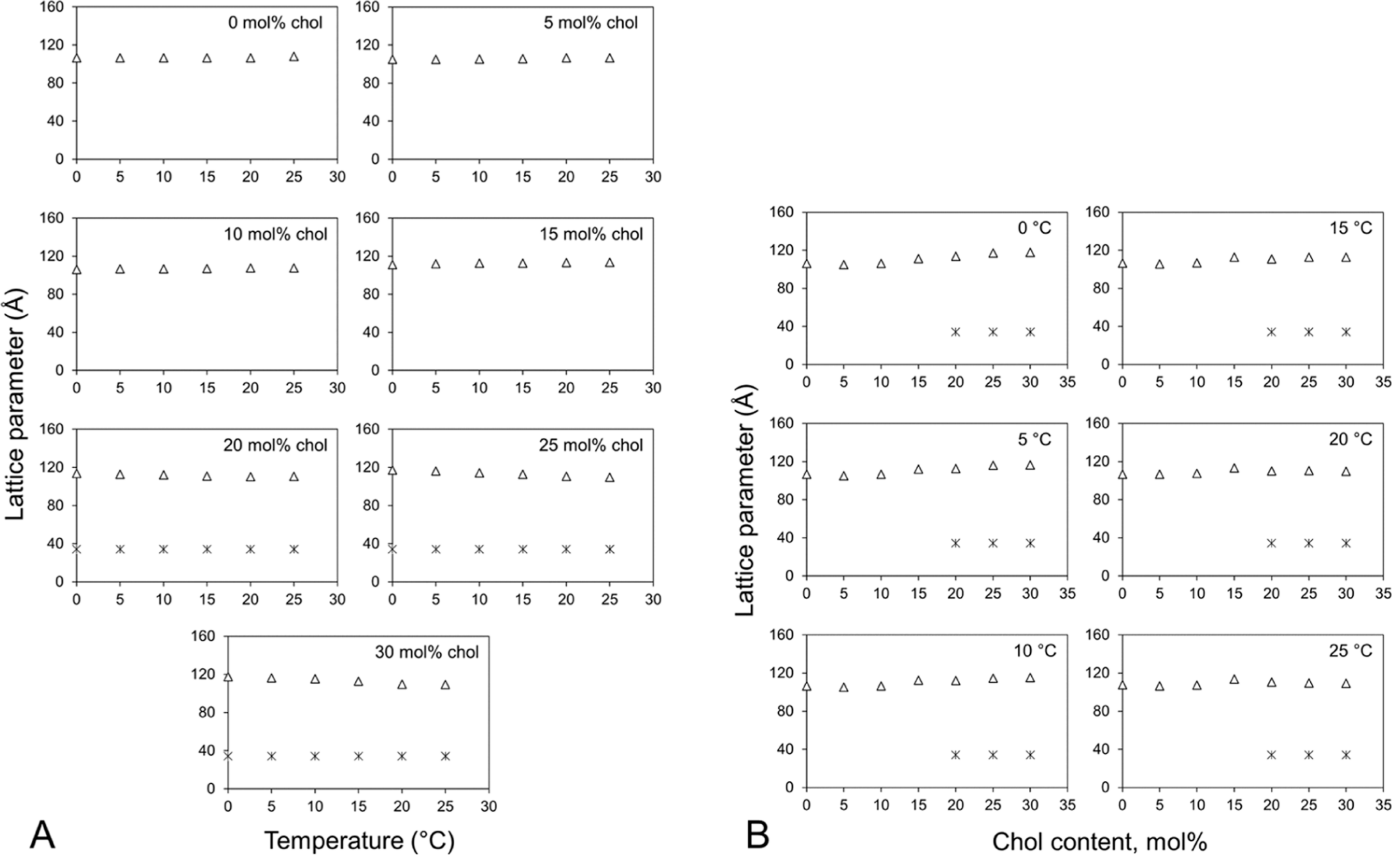
Compatibility of the lipid cubic phase formed by 7.10 MAG with cholesterol. Mixtures of 7.10 MAG and cholesterol were used to form mesophase samples with a water content of 60 (w/w) at 20 °C. Following SAXS measurements for phase identification and microstructure determination at 25 °C, the samples were cooled and incubated at 20, 15, 10, 5, and 0 °C for 30 min before being used for separate rounds of SAXS measurements. Phase lattice parameters are shown as a function of temperature in the cooling direction (A) and as a function of cholesterol concentration (B). The identity of each of the phases is as follows: (Δ) cubic-*Pn*3*m*, and (x) crystalline cholesterol.

**Figure 10:**
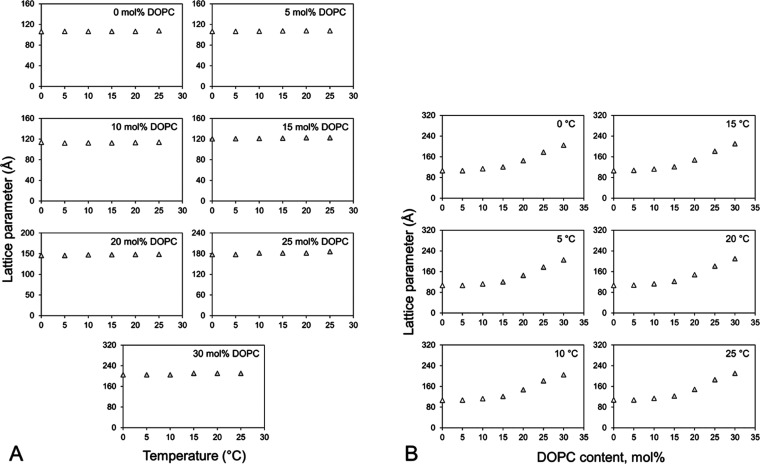
Compatibility of the lipid cubic phase formed by 7.10 MAG with DOPC. Mixtures of 7.10 MAG and DOPC were used to form mesophase samples with a water content of 60% (w/w) at 20 °C. Following SAXS measurements for phase identification and microstructure determination at 25 °C, the samples were cooled and incubated at 20, 15, 10, 5, and 0 °C for 30 min before being used for separate rounds of SAXS measurements. Phase lattice parameters are shown as a function of temperature in the cooling direction (A) and as a function of cholesterol concentration (B). The identity of each of the phases is as follows: (Δ) cubic-*Pn*3*m*.

The host lipid that creates the cubic phase in which crystallization occurs is a MAG. Other lipid types are often combined with the host MAG to facilitate crystal growth. The beneficial effect of these additive lipids can be to replace or substitute for natural lipids that usually associate with the target protein in the native membrane but are lost during the purification process. They may also have an effect by changing the microstructure of the mesophase in a way that ideally facilitates crystal growth. While the cubic mesophase is tolerant to an array of additive lipids and other substances such as detergents, too much of any additive can destabilize the system. In this study, we examined the carrying capacity of the cubic phase formed by hydrated 7.10 MAG for cholesterol and DOPC, two lipids that have been used as additives to facilitate crystallogenesis in the past.^42−44^ Samples were prepared under conditions of full hydration [60% (w/w)] at cholesterol and DOPC concentrations from 0 to 30 mol %. SAXS measurements were made in the temperature range from 25 to 0 °C in 5 °C intervals. The results are included in Figures 9 and 10.

The data show that cholesterol is compatible with the cubic-*Pn*3*m* phase over the entire concentration and temperature ranges examined (Figure 9). Further, the lattice parameter remained constant at ∼110 Å from 0 to 30 mol % cholesterol at 25 °C. However, at and above 20 mol % cholesterol at all temperatures, some of the sterol separates out from the bulk cubic mesophase, with which it coexists in equilibrium, as cholesterol monohydrate. The behavior of 7.10 MAG in its interactions with cholesterol is analogous to that observed with other MAGs such as monoolein and 9.8 MAG.^39,47^

As was observed with cholesterol, DOPC is compatible with 7.10 MAG over the entire range of concentrations and temperatures studied, with most samples remaining in the cubic-*Pn*3*m* phase (Figure 10). However, for some, especially at the higher DOPC concentrations, small amounts of some unidentified phase were present. What is notable about DOPC as an additive lipid is the fact that it causes the lattice parameter of the cubic-*Pn*3*m* phase to rise and to do so quite dramatically starting at ∼15 mol %. Thus, in going from 0 to 30 mol %, the highest DOPC concentration examined, the lattice parameter almost doubles (from 108 Å at 0 mol % to 210 Å at 30 mol % at 25 °C). This swelling effect of DOPC has been observed before with monoolein.^47^ It can be used to facilitate crystallogenesis, as discussed below.

### 3.3 Compatibility of the 7.10 MAG Cubic Phase with Test Membrane Proteins

**Figure 11:**
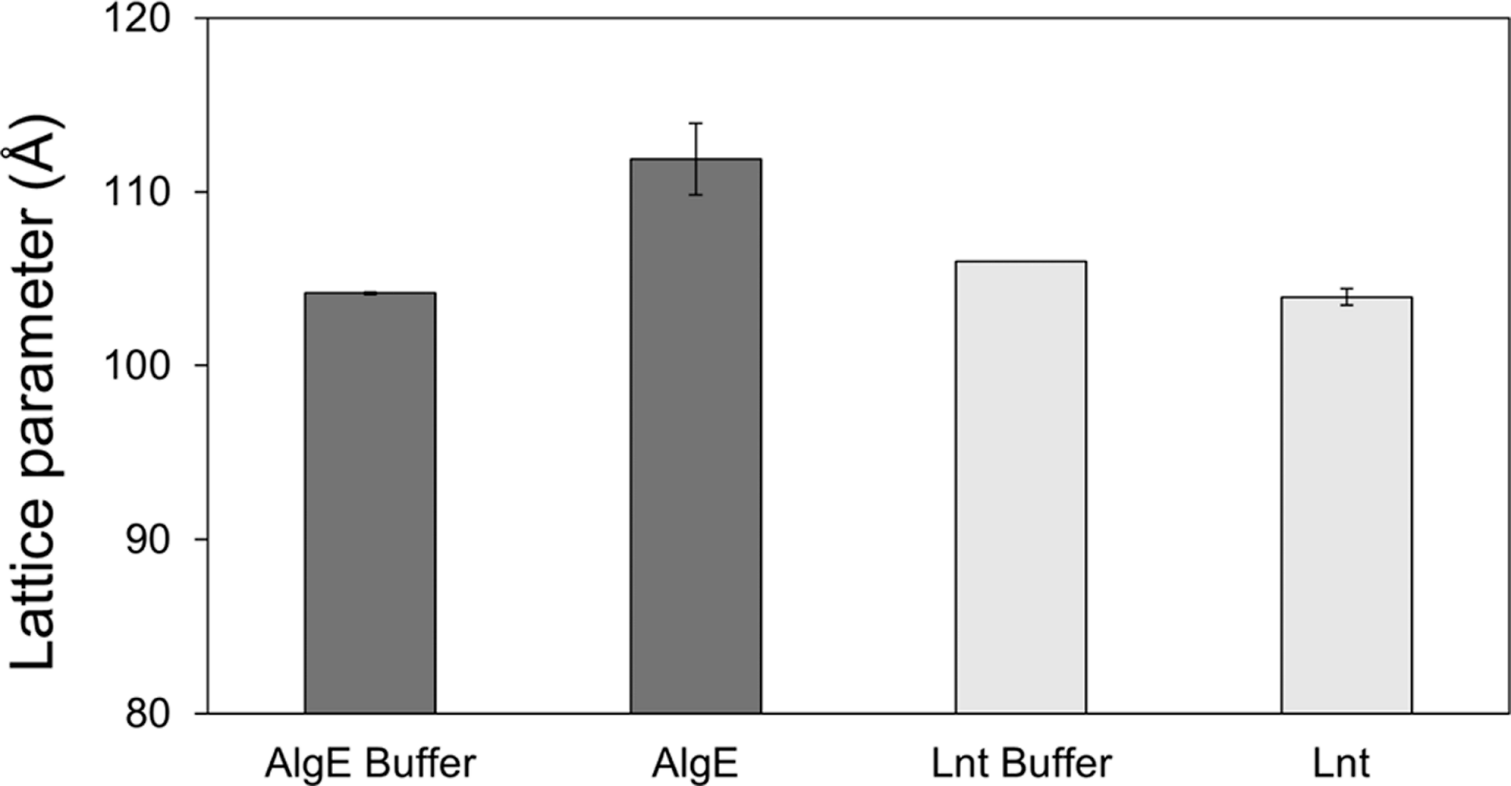
Effect of AlgE and Lnt and their solubilizing buffers on the lattice parameter of the cubic-*Pn3m* phase formed when combined with 7.10 MAG. The lattice parameters are the average of measurements made on duplicate samples.

The compatibility of the test proteins, Lnt and AlgE, with the cubic phase of 7.10 MAG was investigated.^64^ Duplicate samples were prepared as for crystallization trials and subjected to SAXS measurement at 25 °C. The buffers in which the proteins were dissolved were examined first. Buffer A [20 mM sodium citrate pH 6.0, 200 mM NaCl, 10% (v/v) glycerol, 0.05% (w/v) LMNG] and Buffer B [20 mM Tris–HCl pH 8.0, 150 mM NaCl, 0.45% (v/v) C8E4] both gave rise to the cubic-*Pn*3*m* phase with lattice parameters of 106.0 and 104.3 Å which are similar to the value observed when the 7.10 MAG cubic phase is prepared with water (Figure 11). Lnt had no significant effect on the phase behavior stabilizing the cubic-*Pn*3*m* phase with a lattice parameter of 104.0 Å. By contrast, AlgE gave rise to a sample in which the cubic-*Pn*3*m* phase with a lattice parameter of 111.6 Å dominated. A small amount of a second phase, likely the cubic-*Im*3*m* phase, was present in the AlgE-containing sample. It is not clear what accounts for these small differences in phase behavior in the presence of AlgE. However, we do note that the protein concentration used with AlgE is relatively high at 35 mg/mL. Lnt, by comparison, was used at 13 mg/mL. Regardless, both were crystallizable by the *in meso* method in 7.10 MAG.

### 3.4 *In Meso* Crystallogenesis and Structure Determination

One of the principal uses of *N*.*T* MAGs is to grow crystals of membrane proteins for structure determination by X-ray crystallography. Given the mesophase characteristics of 7.10 MAG reported above and its compatibility with additive lipids and membrane proteins, the next logical step was to determine its ability to support crystal growth by the *in meso* method. This was investigated by using three integral membrane proteins. The first was the alginate transporter, AlgE, an 18-stranded β-barrel protein with a molecular weight of 51 kDa that resides in the outer membrane of *P. aeruginosa*.^49,60^ The second is the lipoprotein *N*-acyltransferase, Lnt, from *E. coli*.^48,65^ Lnt has half its 57 kDa mass as an α-helical bundle residing in the cytoplasmic membrane, on top of which sits a domain that is a mix of β-strands and α-helices in the form of an αββα sandwich. Finally, the predominantly α-helical adenosine receptor, A2aR,^50^ which requires the addition of cholesterol to the mesophase for growth of diffraction quality crystals, was tested.

**Figure 12:**
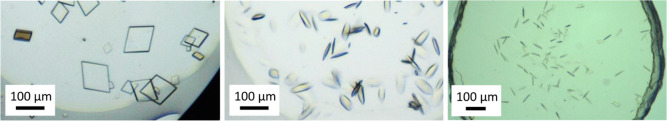
Crystals of AlgE (left), Lnt (middle) and A2aR (right) grown in 7.10 MAG at 20 °C. AlgE crystals, grown in 100 mM sodium citrate pH 5.6, 25–300 mM (NH_4_)_2_SO_4_ and 34–41% (v/v) PEG400 for 30 days show the usual ziggurat arrangement. Lnt crystals, grown for 30 days in 100 mM MES at pH 6.0, 8% (v/v) MPD, and either sodium or potassium thiocyanate from 50 to 400 mM, also display the expected bullet or rice shape. A2aR crystals grew as needles in 30–35.5% (v/v) PEG400, 50 mM sodium thiocyanate, 100 mM sodium citrate at pH 5, 0.2% (v/v) 2,5-hexanediol, and 25 μM ZM241385 and were harvested after 14 days.

**Figure 13:**
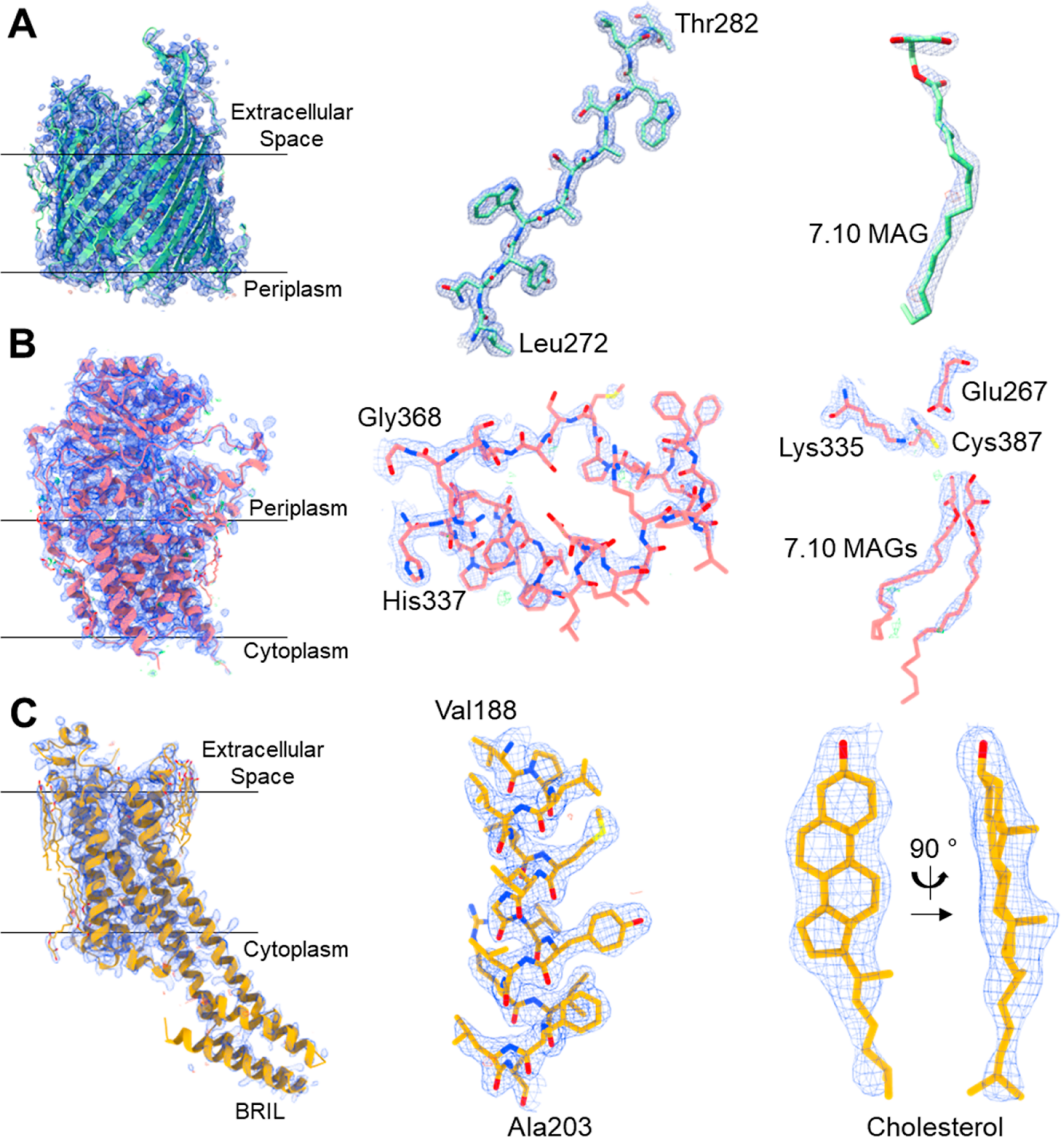
X-ray structures and corresponding electron density of AlgE, Lnt and A2aR obtained using crystals grown by the *in meso* method in 7.10 MAG. Electron density is displayed in mesh representation as follows: blue mesh––2Fo-Fc map contoured at 1.5 σ; green mesh––Fo-Fc map contoured at 3 σ; and red mesh––Fo-Fc map contoured at −3 σ. All maps are carved 1.6 Å from the shown atoms. (A) Left: AlgE structure. Middle: β-Strand L272-T282. Right: A 7.10 MAG molecule. (B) Left: Lnt structure. Middle: Arm 3 (residues H337-G368). Right: active site residues E267, K335 and C387, with two 7.10 MAG molecules in the binding pocket. (C) Left: A2aR-BRIL structure. Middle: α-Helix V188-A203. Right: cholesterol molecule bound to A2aR in two views 90° apart.

Crystals of AlgE grew readily as thin plates at 20 °C in the cubic phase formed by 7.10 MAG (Figure 12). The crystals displayed anisotropic diffraction to 1.45 Å resolution with electron density of excellent quality enabling the modeling into density of 459 out of 479 residues in the protein (Figure 13A). Two stretches of the protein were disordered (109–117, 440–449) corresponding to loops on either end of the barrel. The overall fold of the protein is similar to what has been reported previously (root-mean-square deviation (rmsd) value of 0.29 Å, relative to PDB code 4AFK).^60^ The hydrophobic surface of the protein is coated with 11 lobes of density that have been modeled as 7.10 MAG. They are configured in a way that is reminiscent of how lipids that make up the bilayer membrane in which the protein resides naturally are arranged. The structure includes calcium and sodium atoms as well as sulfate and citrate ions. These have been seen before and are found in the expected locations. The citrate sits in the core of the barrel and is taken as a mimic for the mannuronate and guluronate components of the alginate copolymer. As was observed with AlgE crystallized in 9.8 MAG, the hexahistidine N-terminus folds into the core of the barrel where it associates with the citrate and sulfate ions.^39^

Compared to AlgE, Lnt performed equally well in 7.10 MAG with crystals measuring ∼50 μm in maximum dimension growing in 4 days at 20 °C (Figure 12). The structure (Figure 13B), obtained by molecular replacement to a resolution of 2.19 Å, was of very good quality with just two residues (235–236) that could not be placed in reliable density. These reside in the extracellular loop that coordinates with the phosphate in the glycerophospholipid substrate and product and that is known to be flexible.^65^ Eight structured lipids were modeled into density as 7.10 MAG molecules. Six decorate the outer surface of the transmembrane helical core of Lnt, roughly in a bilayer arrangement as observed with AlgE. Two MAGs are found stacked one behind the other in the binding pocket leading to the active site and are taken to mimic the acyl chains in the glycerophospholipid and lipoprotein substrates and products. The catalytic Cys387 is natively acylated. However, no such acylation was apparent in the solved structure, suggesting that the chain had been hydrolyzed during protein purification and/or crystallization. Arm 3 is a flexible loop (His337-Gly368) that helps secure substrate molecules in the binding pocket of the enzyme and is often not seen in density. In the current structure, good density is present for essentially all of the backbone and most of the side chains in this long loop. Several residues (Pro340, Phe341, Gly342, and Pro346) in the loop are found stabilizing the 7.10 MAGs in the binding pocket. Crystal packing gives rise to an arrangement wherein the N-terminal transmembrane helix of one Lnt molecule sits between Arm1 and Arm3 of a second Lnt molecule, further stabilizing Arm 3 and contributing to the defined density for this usually flexible region (Figure S6). This likely mimics the way Lnt lipoprotein substrates that have transmembrane helices, such as the SlyB component of the PhoPQ regulon,^66^ engage with the enzyme as a prelude to *N*-acylation. Undoubtedly, close contact with the *N*-terminal helix contributes to the ordering of Arm 3.

GPCRs require the addition of cholesterol to the host MAG for the growth of diffraction quality crystals by the *in meso* method. Upon noting that 7.10 MAG was able to accommodate cholesterol to 20 mol %, we sought to test its usefulness in crystallizing a benchmark GPCR, A2aR. Crystals grew to ∼50 μm in 14 days and displayed anisotropic diffraction to 2.4 Å resolution. The solved structure is shown in Figure 13C. The construct, which has the BRIL fusion protein inserted in the third intracellular loop, has excellent density for the entire receptor. The electron density surrounding the BRIL is less clear, with very high *B*-factor values relative to receptor residues. The receptor is decorated with nine molecules of 7.10 MAG arranged in a similar bilayer arrangement seen for AlgE and Lnt, as well as three well-ordered cholesterol molecules. The antagonist, ZM241385, added during purification and in the precipitant screen, is seen bound in the extracellular ligand binding pocket. The protein fold is highly similar to previously solved structures (rmsd value of 0.32 Å compared to PDB code 4EIY).^50^

## 4 Discussion

The temperature and hydration-dependence of the phases formed by a new lipid, 7.10 MAG, have been established by SAXS/WAXS under operational and approach-to-equilibrium conditions. At full hydration, the cubic phase of 7.10 MAG accommodates both cholesterol and DOPC as additive lipids over wide limits and supports crystallization by the *in meso* method of three quite distinct membrane proteins, AlgE, Lnt, and A2aR, facilitating their structure determination by crystallographic means to high resolution. 7.10 MAG can now be included in a host MAG lipid screen to optimize the crystallization of membrane proteins. It might also be used in combination with other MAGs to modify their phase behavior and microstructure as part of the crystallization optimization process. Relatedly, mixtures of 1–9.9 MAG and 2–9.9 MAG have been used successfully to crystallize and to determine the structures of AlgE and Lnt.^67^

7.10 MAG is member of a series of closely related cis monounsaturated MAGs whose *T*–*C* phase diagrams have been constructed using SAXS/WAXS.^11,18,19,31−41^ Common to most is the presence of solid, Lc, liquid, FI, and several liquid-crystalline phases to include the L_α_, cubic-*Ia*3*d* and cubic-*Pn*3*m* phases. The H_II_ phase was not observed in 7.10 MAG, nor indeed does it appear in the case of 7.9 MAG or 9.7 MAG.^31,35^ The H_II_ phase is a mesophase that forms fully hydrated at relatively low water contents and is associated with amphiphiles that adopt a dynamically averaged wedge shape for close packing into long cylinders. The polar end of the MAG molecule occupies the small end of the wedge, where it is in contact with the aqueous core of the cylinder. The acyl chain is increasingly disordered toward its methyl terminus, creating the wider end of the wedge. In the *T* = 9 series of MAGs, H_II_ becomes apparent when *N* is equal to or greater than 9.^34,37,38,40^ It is the dominant phase in 13.9 MAG.^37^ By contrast, it is not present in 7.9 MAG.^35^ Nor indeed is it observed with 7.10 MAG, as noted. Thus, whether a particular phase is expressed or not depends sensitively on the detailed chemical makeup of the acyl chain. Changing the position of the double bond by just one carbon or changing *N* or *T* by a single methylene can have major consequences in terms of phase behavior and, by extension, on the phase microstructure and the functionality of the corresponding lipid.

It is important to note that these observations regarding phase behavior refer to MAGs hydrated with water in the absence of other aqueous additives. By contrast, small amounts of salt in the aqueous solution can profoundly affect phase properties. In the case of 9.9 MAG, the H_II_ phase is a relatively minor phase that appears at high temperatures. It becomes the dominant phase that is stable to lower temperatures at high concentrations of sodium chloride.^7^ Remarkably, monoelaidin, which is 9.9 MAG with a trans instead of a cis olefinic bond at carbon number 9, does not form the H_II_ phase in pure water. However, small amounts of sodium chloride induce H_II_ phase expression. As with 9.9 MAG, H_II_ becomes the dominant phase at high salt concentrations.^7^

The crystal structures of AlgE and Lnt obtained using 7.10 MAG are, for the most part, superimposable on those generated using 9.9 MAG.^39,48,60,65^ It is very likely that the bilayer thickness and fluidity characteristics of the hosting mesophase formed in these different MAGs and used to grow crystals are slightly different. However, these differences do not show up in the corresponding crystal structures, suggesting that the final form adopted in the crystal reflects the state of the protein assumed in the native membrane which is unlikely to change dramatically in the course of the protein’s activity cycle. However, with other proteins such as the mechanosensitive ion channels where adjustments in shape and orientation in the membrane are part of the mechanism of action,^68^ these different MAGs might be used to advantage to capture different states in the cycle.

Undercooling of the liquid-crystalline phases was observed in the operational phase diagram of 7.10 MAG. This is a characteristic of the many *N*.*T* MAGs that have been studied to date.^19,33,34,38,39^ Undercooling is a desirable characteristic, especially since the undercooled state can persist for extended periods. This means that crystallization trials can be carried out at low temperatures, typically 4 °C, where the protein is likely to be more stable. Some membrane proteins are robust and can be handled and used in successful crystallization trials that can last for months. However, others are labile and must be worked on in ice/water to minimize inactivation. In such cases, the ability to carry out crystallization trials at 4 °C or below is a big advantage. It seems likely too that stability is engendered in the process of reconstituting the protein out of an unnatural detergent micelle and into a more native-like bilayer of the mesophase as part of the *in meso* crystallization process. By lowering the temperature, the microstructure of the mesophase can change. This provides an additional screening tool with which to optimize crystal growth. Accordingly, a particular protein may crystallize at 4 °C but not at RT. Being able to use the temperature over a wide range as a screening variable is enormously beneficial. As shown in the phase diagrams reported in this study, the cubic phase of 7.10 MAG is accessible for use in screens over an impressive 100 °C range all the way from −5 to 95 °C.

To be optimally useful in crystallization screening trials, the host lipid must be compatible with a variety of additives. Other lipids are often used as additives to facilitate crystallogenesis and the cubic phase has proven to be compatible with a number of them including glycerophospholipids and sterols.^39,47^ In the current study, 7.10 MAG has been shown to accommodate cholesterol and DOPC. Both lipids have been used to advantage in the crystallization and structure determination of membrane proteins. Cholesterol, in particular, is now used routinely as an additive in *in meso* crystal structure work involving GPCRs and the complexes they form.^69^ Cholesterol has minimal effect on the microstructure of the cubic-*Pn*3*m* phase up to a concentration of ∼20 mol % (Figure 9). Beyond this, crystals of the sterol monohydrate separate from a bulk-cholesterol-saturated cubic phase. Phase behavior with cholesterol was insensitive to temperature from 0 to 25 °C, which means it can be used as an additive in trials conducted in this temperature range.

The test glycerophospholipid used in this study was DOPC. It was found to be compatible with the cubic phase of 7.10 MAG, as observed with 9.9 MAG (Figure 10). Thus, the cubic-Pn3m phase persisted in the presence of 30 mol % DOPC, and it did so in the range from 0 to 25 °C. Interestingly, the cubic phase underwent a dramatic rise in lattice parameter with increasing DOPC concentration, effectively doubling in the concentration range studied. DOPC can be viewed as having a dynamically averaged cylindrical shape. When combined in the cubic phase with 7.10 MAG, which is wedge-shaped, it has the effect of reducing curvature at the lipid/water interface, causing the mesophase to imbibe more water and the lattice parameter to rise. It is apparent therefore that in addition to using PC to augment important native membrane lipids that may be lost during purification, it can also be used to craft a mesophase with large aqueous channels needed to accommodate bulky extramembrane domains in certain target proteins and complexes. Such swollen mesophases have been used to advantage in the crystallization and structure determination of the adrenoreceptor–Gs protein and rhodopsin–arrestin complexes, both of which have extensive extramembrane parts.^42,70^ Swollen cubic phases can also be accessed using charged lipids as additives as is the case with phosphatidylserine and cardiolipin.^47^

This study contributes the phase behavior of another *N*.*T* MAG to a growing database of related information. Phase behavior takes the form of temperature–composition phase diagrams covering a wide range of temperatures and hydration recorded under operational and approach-to-equilibrium conditions. With data collected using SAXS/WAXS, quantitative phase microstructure information is available for all samples as a function of temperature and composition. It is now possible to analyze the data and to identify relationships between the chemical makeup of a MAG and its corresponding phase behavior and microstructure of the type referred to in Introduction. The information can be exploited to design novel MAGs with desirable characteristics. At its simplest, this can be done by extrapolating and interpolating phase transitions and boundaries beyond and between the phase diagrams in a series of MAGs. This approach has paid dividends in the design of 7.7 MAG and 9.7 MAG for use with proteins that have large extramembrane domains.^36,42,70,71^ In a very different application, 7.9 MAG was crafted to have a low Lc-to-cubic phase transition temperature under conditions of full hydration suited for use in crystallizing thermally sensitive membrane proteins.^35^ For this purpose, extrapolations of transition temperatures and compositions in the corresponding phase diagrams across a “*T* = 9” series of four MAGs (9.9 MAG, 10.9 MAG, 11.9 MAG, and 13.9 MAG) were used. Adding the 7.10 MAG phase information, as generated in this study, to the extant database will contribute to making these predictions more reliable. Longer term, the goal is to mine the database for a more rational approach to designing *N*.*T* MAGs with the desired properties. This will be tackled using a combination of artificial intelligence, machine learning in particular, and molecular dynamics simulations to develop algorithms that can identify specific *N*.*T* MAGs with a distinct mesophase behavior and microstructure for defined downstream applications. The lipids can be synthesized and characterized using SAXS/WAXS. In turn, the new information can be added to the database to further refine and extend the rational design approach.^72^

## Supporting Information Available

7.10 MAG synthesis pathway and quality characterization of 7.10 MAG (PDF)
Movie describing sample preparation for SAXS/WAXS and capillary loading and sealing
(MP4)

## Abbreviations

A2aR: adenosine A2a G protein-coupled receptor
C8E4: *n*-octyl tetraoxyethylene
CHS: cholesteryl hemisuccinate
DDM: *n*-dodecyl-β-d-maltoside
DM: *n*-decyl-β-d-maltopyranoside
DLS: Diamond Light Source
EDTA: ethylenediaminetetraacetic acid
ESI: electrospray ionization
FI: fluid isotropic phase
FPLC: fast protein liquid chromatography
GPCR: G protein-coupled receptor
HEPES: 4-(2-hydroxyethyl)-1-piperazineethanesulfonic acid
H_II_: inverted hexagonal phase
IPTG: isopropyl β-d-1-thiogalactopyranoside
L_α_: lamellar liquid-crystalline phase
LB: Luria broth
Lc: lamellar crystalline phase
LCP: lipid cubic phase
LMNG: 2,2-didecylpropane-1,3-bis-β-d-maltopyranoside
MAG: monoacylglycerol
MES: 2-(*N*-morpholino)ethanesulfonic acid
MPD: 2-methyl-2,4-pentanediol
NMR: nuclear magnetic resonance
PEG400: polyethylene glycol 400
PMSF: phenylmethylsulfonyl fluoride
SAXS: small-angle X-ray scattering
SDS: sodium dodecyl sulfate
SLS: Swiss Light Source
*T*–*C*: temperature–composition
TLC: thin-layer chromatography
Tris: tris(hydroxymethyl)aminomethane
UV: ultraviolet
WAXS: wide-angle X-ray scattering
WT: wild-type
