# Supplementary material for: 7.10 MAG. A Novel Host Monoacylglyceride for In Meso (Lipid Cubic Phase) Crystallization of Membrane Proteins
Source: Cryst Growth Des. 2024 Mar 25;24(7):2985–3001. doi: 10.1021/acs.cgd.4c00087 (PMC10995948; doi:10.1021/acs.cgd.4c00087)
Supplement: Supplementary file 1 — cg4c00087_si_001.pdf [file cg4c00087_si_001.pdf]

Supplementary Information

7.10 MAG. A new host lipid for *in meso* (lipid cubic phase) crystallization of integral membrane proteins

Pawel Krawinski [a§], Luke Smithers [a§], Leendert van Dalsen [a,b], Coilin Boland [a], Nikita Ostrovitsa [b], Javier Pérez [c], and Martin Caffrey [a*]

[a] Membrane Structural and Functional Biology Group, School of Medicine and School of Biochemistry and Immunology, Trinity College Dublin, Dublin D02 R590, Ireland.

[b] School of Chemistry, Trinity College Dublin, Dublin D02 R590, Ireland.

[c] SWING Beamline, Synchrotron Soleil, 91190 Saint-Aubin, France.

Correspondence e-mail: martin.caffrey@tcd.ie

7.10 MAG Synthesis and Purification

General procedure. The procedure followed in the current study is a slight modification of a published method.[27,28] Reagents were purchased from commercial sources and used without further purification. Thin layer chromatography analysis was performed using Merck F_{254} silica gel 60 plates (175 – 225 µm thickness). Nuclear magnetic resonance (NMR) spectra were obtained with a Bruker Avance 400 spectrometer. ^{1}H NMR (400.13 MHz) were recorded in $CDCl_3$ relative to the residual solvent signal (δ_H = 7.26 ppm) unless otherwise specified; coupling constants are reported in Hz. ^{13}C NMR (100.6 MHz) were recorded in $CDCl_3$ relative to the residual solvent signal (δ_C = 77.2 ppm) unless otherwise specified. Chemical shifts are reported in parts per million (ppm, δ) downfield from tetramethylsilane. Mass spectra were recorded using an electrospray ionisation (ESI) ion-trap on a Bruker micrOTOF-Q III spectrometer interfaced to a Dionex UltiMate 3000 LC in positive and negative modes as required. Unless otherwise indicated procedures were carried out at room temperature which varied from 19.5 to 20.5 °C.

Synthesis of 7.10 MAG

Scheme 1. Synthesis pathway

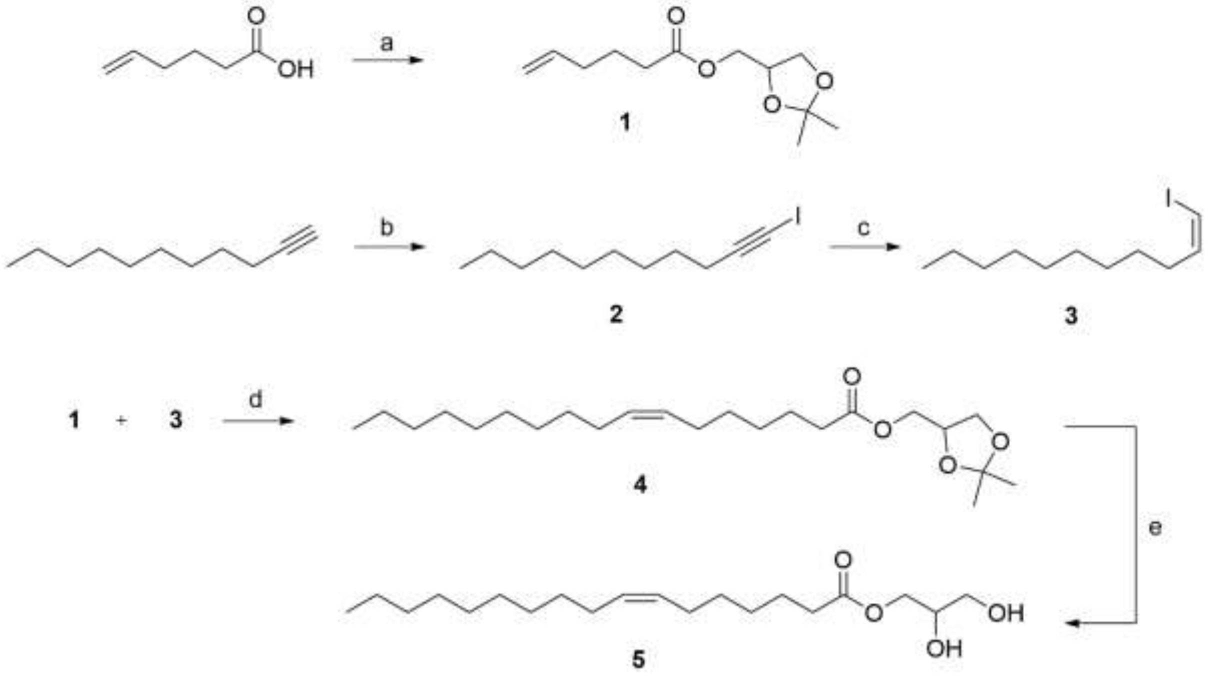

Reaction conditions: (a) DMAP, DCC, solketal, CH_2Cl_2; **(b)** I_2, TBAB, CuI, NEt_3, H_2O; **(c)** BH_3.DMS, cyclohexene, Et_2O, AcOH; **(d) i)** 9-BBN, THF; **ii) 3**, $AsPh_3$, Cs_2CO_3, Pd(dppf)Cl_2.CH_2Cl_2, DMF, H_2O; **(e)** AcOH, H_2O.

Synthesis of (2,2-dimethyl-1,3-dioxolan-4-yl)methyl hex-5-enoate

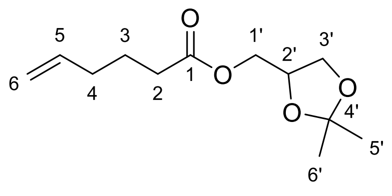

In an oven-dried 500 mL round bottom flask, 4-dimethylaminopyridine (7.33 g, 60.0 mmol, 1.0 eq.) was dissolved in anhydrous dichloromethane (100 mL). Hex-5-enoic acid (6.85 g, 60.0 mmol, 1.0 eq.)

and solketal (8.72 g, 66.0 mmol, 1.1 eq.) were added sequentially, via syringe, under a positive pressure of argon. The solution was brought to 0 °C and a solution of *N*,*N'*-dicyclohexylcarbodiimide (13.62 g, 66.0 mmol, 1.1 eq.) in anhydrous dichloromethane (50 mL) was added dropwise over 10 min. The reaction was allowed to heat to 20 °C and stirred for 15 h. The resulting mixture was cooled to -20 °C and the precipitate was filtered by vacuum filtration. The filtrate was concentrated under vacuum and purified by column chromatography over silica gel, eluting with hexanes:ethyl acetate (7:3 by vol.) to yield the product as a colourless oil (9.59 g, 71%).

^{1}H-NMR ($CDCl_3$, 400 MHz, ppm): δ = 1.34 (s, 3H, H6'), 1.41 (s, 3H, H5'), 1.66-1.76 (m, 2H, H3), 2.04-2.10 (m, 2H, H4), 2.33 (m, 2H, H2), 3.69-3.73 (dd, J_1 = 8.44 Hz, J_2 = 6.18 Hz, 1H, H3'), 4.03-4.16 (m, 3H, H1', H3'), 4.26-4.32 (m, 1H, H2'), 4.94-5.03 (m, 2H, H6), 5.69-5.80 (m, 1H, H5).

^{13}C-NMR ($CDCl_3$, 100 MHz, ppm): δ = 23.91, 25.33, 26.64, 32.96, 33.27, 64.52, 66.29, 73.59, 109.76, 115.39, 137.51, 173.28.

Synthesis of 1-iodoundec-1-yne

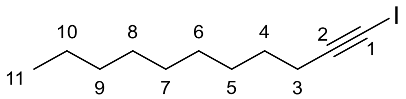

To a 500 mL round-bottom flask charged with deionised water (175 mL) was added tetra-*n*-butylammonium bromide (16.30 g, 50.56 mmol, 1.0 eq.) and copper iodide (0.10 g, 0.51 mmol, 0.01 eq.) and the mixture stirred vigorously for 10 min. Iodine (25.67 g, 101.12 mmol, 2.0 eq.) was added, followed by triethylamine (21 mL, 151.68 mmol, 3.0 eq.) added via syringe and the mixture stirred vigorously for 10 min. Undec-1-yne (10 mL, 50.56 mmol, 1.0 eq.) was added via syringe and the mixture stirred vigorously for 23 h. The product was extracted with ethyl acetate (3 x 300 mL), the combined organic layers were washed with saturated aqueous sodium thiosulfate (3 x 200 mL) and saturated aqueous sodium chloride (200 mL), dried over magnesium sulfate, and concentrated under vacuum. The crude material was purified by column chromatography over silica gel, eluting with 100% hexanes to yield the product as a pale-yellow oil (12.67 g, 90%).

^{1}H-NMR ($CDCl_3$, 400 MHz, ppm): δ = 0.88 (m 3H, H11), 1.21-1.40 (m, 12H, H5, H6, H7, H8, H9, H10), 1.47-1.55 (m, 2H, H4), 2.35 (t, J = 7.10 Hz, 2H, H3).

^{13}C-NMR ($CDCl_3$, 100 MHz, ppm): δ = -7.54, 14.28, 20.99, 22.85, 28.66, 28.95, 29.25, 29.43, 29.61, 32.04, 95.05.

Synthesis of (*Z*) 1-iodoundec-1-ene

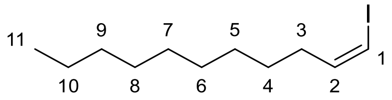

To an oven-dried 250 mL 3-neck round bottom flask charged with anhydrous diethyl ether (60 mL), was added borane dimethyl sulphide complex (2 M in tetrahydrofuran, 25.1 mL, 50.13 mmol, 1.1 eq.) via syringe under argon. The solution was cooled to 0 °C and anhydrous cyclohexene (11.2 mL, 110.27 mmol, 2.2 eq.) was added dropwise via syringe over 10 min. The mixture was stirred at 0 °C for 15 min, heated to 20 °C and stirred for 1 h. The mixture was cooled to 0 °C and 1-iodoundec-1-yne (12.66 g, 45.57 mmol, 1.0 eq.) added dropwise via syringe. The mixture was stirred at 0 °C for 30 min, heated to 20 °C and stirred for 2 h. The mixture was cooled to 0 °C and acetic acid (42 mL, 16.0 eq.) added via syringe over 15 min, the mixture heated to 20 °C and stirred for 2.5 h. The mixture was diluted with diethyl ether (60 mL) and deionised water (80 mL), and the organic layer washed

with deionised water (2 x 100 mL) and saturated aqueous sodium chloride (2 x 100 mL), dried over magnesium sulfate, swirled with ethanolamine (30 mL) and the resulting white precipitate filtered. The filtrate was concentrated under vacuum and purified by column chromatography over silica gel, eluting with 100% hexane to yield a pale-yellow oil (7.70 g, 60%)

^{1}H-NMR ($CDCl_3$, 400 MHz, ppm): δ = 0.88 (m 3H, H11), 1.22-1.46 (m, 14H, H4, H5, H6, H7, H8, H9, H10), 2.11-2.17 (m 2H, H3), 6.13-6.20 (m, 2H, H1, H2).

^{13}C-NMR ($CDCl_3$, 100 MHz, ppm): δ = 14.29, 21.08, 22.88, 28.68, 28.95, 29.25, 29.45, 29.62, 32.05, 82.27, 141.67.

Synthesis of (2,2-dimethyl-1,3-dioxolan-4-yl)methyl (*Z*) septadec-7-enoate

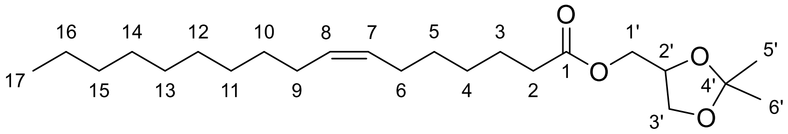

To an oven-dried 500 mL 3-neck round bottom flask charged with anhydrous tetrahydrofuran (75 mL) was added (2,2-dimethyl-1,3-dioxolan-4-yl)methyl hex-5-enoate (5.70 g, 24.98 mmol, 1.0 eq.). The solution was cooled to 0 °C and 9-borabicyclo[3.3. 1]nonane (0.5 M in tetrahydrofuran, 55.0 mL, 27.48 mmol, 1.1 eq.) added dropwise via syringe. The reaction was allowed to heat to 20 °C and stirred for 19 h. The reaction vessel was then quenched with deionised water (75 mL) for 2 h. To a separate oven-dried round bottom flask charged with dimethylformamide (140 mL) was added triphenylarsine (1.68 g, 5.50 mmol, 0.2 eq.), caesium carbonate (12.21 g, 37.47 mmol, 1.5 eq.) and (*Z*) 1-iodoundec-1-ene (7.70 g, 27.48 mmol, 1.1 eq.). Both vessels were degassed via the freeze-pump-thaw method and sparged with argon for 15 min. To the vessel containing dimethylformamide was added [1,1'-bis(diphenylphosphino)ferrocene]dichloropalladium.dichloromethane complex (1.12 g, 1.37 mmol, 0.05 eq.) and the solution sparged with argon for 15 min. The tetrahydrofuran mixture was transferred to the dimethylformamide vessel via canula over 15 min and the reaction stirred for 16 h. The mixture was added to saturated aqueous sodium chloride (500 mL), the product extracted with diethyl ether (3 x 300 mL), the combined organic layers were washed with deionised water (200 mL) saturated aqueous sodium chloride (200 mL), dried over magnesium sulfate and concentrated under vacuum. The crude material was purified by column chromatography over silica gel, eluting with hexanes:ethyl acetate (95:5 by vol.) to yield the product as a pale yellow oil (6.19 g, 65%).

^{1}H-NMR ($CDCl_3$, 400 MHz, ppm): δ = 0.87 (m, 3H, H17), 1.21-1.35 (m, 18H, H4, H5, H10, H11, H12, H13, H14, H15, H16), 1.36 (s, 3H, H6'), 1.43 (s, 3H, H5'), 1.58-1.67 (m, 2H, H3), 1.96-2.05 (m, 4H, H6, H9), 2.34 (m, 2H, H2), 3.71-3.75 (m, 1H, H3'), 4.05-4.18 (m, 3H, H1', H3'), 4.27-4.34 (m, 1H, H2'), 5.28-5.39 (m, 2H, H7, H8).

^{13}C-NMR ($CDCl_3$, 100 MHz, ppm): δ = 14.28, 22.85, 24.98, 25.56, 26.86, 27.17, 27.40, 28.93, 29.50, 29.53, 29.74, 29.78, 29.92, 32.08, 34.24, 64.71, 66.53, 73.83, 109.99, 129.58, 130.42, 173.71.

Synthesis of 2,3-dihydroxylpropyl (*Z*) septadec-7-enoate (7.10 MAG)

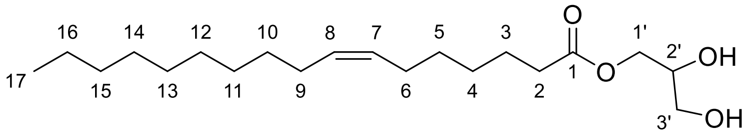

To a 250 mL round bottom flask charged with acetic acid:water (100 mL, 4:1 by vol.) was added (2,2-dimethyl-1,3-dioxolan-4-yl)methyl (*Z*) septadec-7-enoate (3.34 g, 8.75 mmol, 1.0 eq.). The mixture was heated to 50 °C and stirred for 2 h. The mixture was quenched with saturated aqueous sodium bicarbonate to a pH of 7. The product was extracted with diethyl ether (3 x 200 mL), the combined organic layers washed with deionised water (200 mL) and saturated aqueous sodium chloride (200 mL), dried over magnesium sulfate and concentrated under vacuum. The crude was purified by column chromatography over silica gel, eluting with gradient elution from hexanes:ethyl acetate (3:1 by vol.) to hexanes:ethyl acetate:acetone (2:1:1 by vol.) to yield the product as a pale yellow waxy solid (1.63 g, 54 %).

^{1}H-NMR ($CDCl_3$, 400 MHz, ppm): δ = 0.88 (m, 3H, H17), 1.22-1.38 (m, 18H, H4, H5, H10, H11, H12, H13, H14, H15, H16), 1.60-1.69 (m, 2H, H3), 1.97-2.06 (m, 4H, H6, H9), 2.35 (m, 2H, H2), 3.57-3.72 (m, 2H, H3'), 3.90-3.96 (m, 1H, H2'), 4.13-4.24 (m, 2H, H1'), 5.28-5.40 (m, 2H, H7, H8).

^{13}C-NMR ($CDCl_3$, 100 MHz, ppm): δ = 14.29, 22.86, 25.00, 27.16, 27.42, 28.93, 29.52, 29.75, 29.79, 29.93, 32.09, 34.29, 63.51, 65.37, 70.45, 129.53, 130.48, 174.46.

HRMS (m/z ES$^+$): $C_{20}H_{39}O_4$ $[M+H]^+$; Calculated 343.2842, Found 343.2845.

Quality characterization of synthetic 7.10 MAG

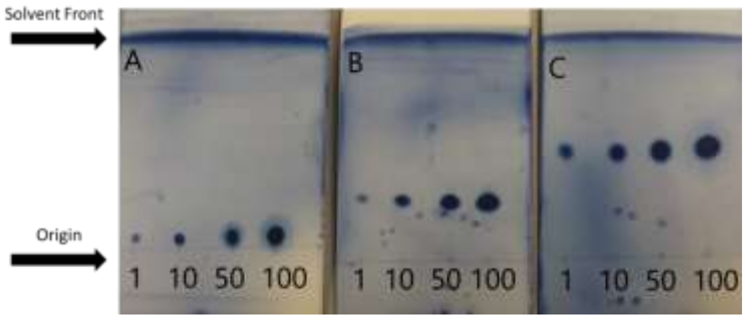

Figure S1. Thin layer chromatographic analysis of synthetic 7.10 MAG. Lipid loadings in micrograms are indicated at the bottom of each lane. Eluting solvent systems are as follows: (**A**) hexanes:ethyl acetate (75:25 by vol.); (**B**) hexanes:acetone:ethyl acetate (74:25:1 by vol.); (**C**) hexanes:acetone:ethyl acetate (50:25:25 by vol.). Lipid was placed on the plate as a solution in hexanes. Spots were visualized by staining with a 0.04 M ammonium molybdate solution followed by charring with a heat gun.

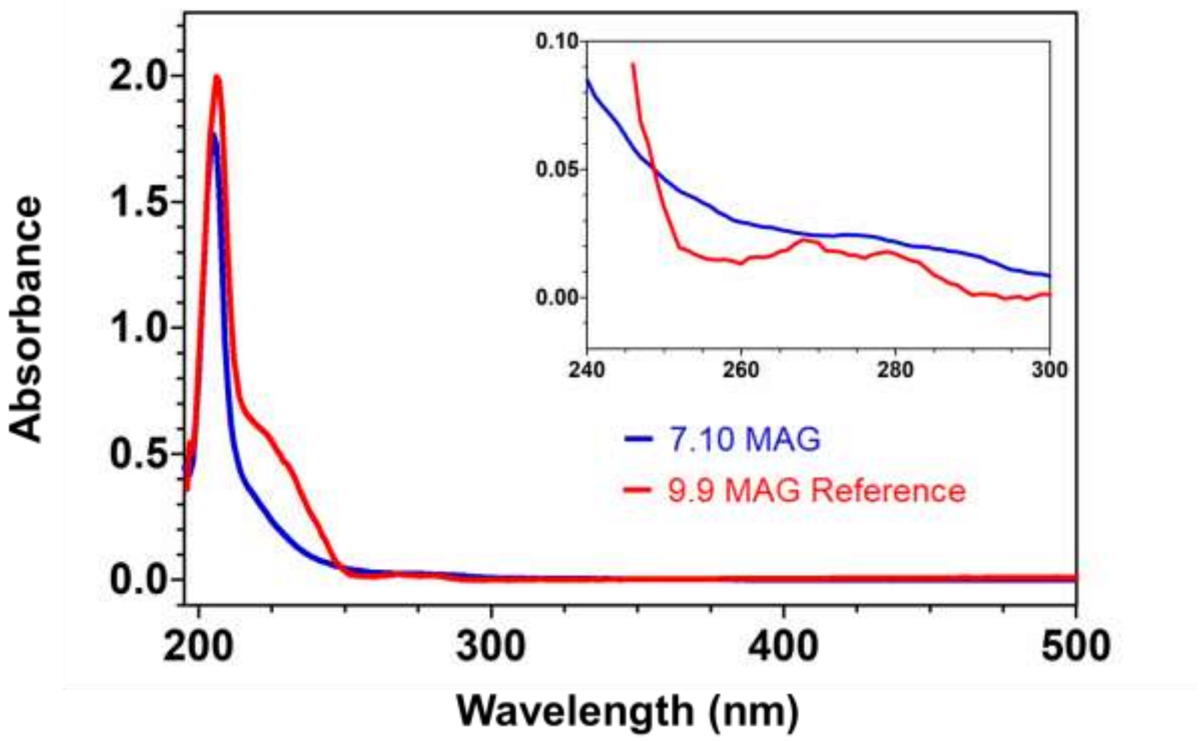

Figure S2. UV-visible absorption spectrum of synthetic 7.10 MAG and commercial 9.9 MAG. Measurements were made in 1 cm quartz cuvettes at a lipid concentration of 14 mM in ethanol. The commercial lipid was obtained from Nu Chek (M239-025-C). Ultraviolet (UV) absorbance by the lipid can impact on its use in spectroscopic measurements of reconstituted membrane proteins in the cubic phase, where the lipid is present at a concentration of ~2 M. Relatedly, trace impurities have recently been identified as contributors to the UV absorption properties of fatty acids.[72]

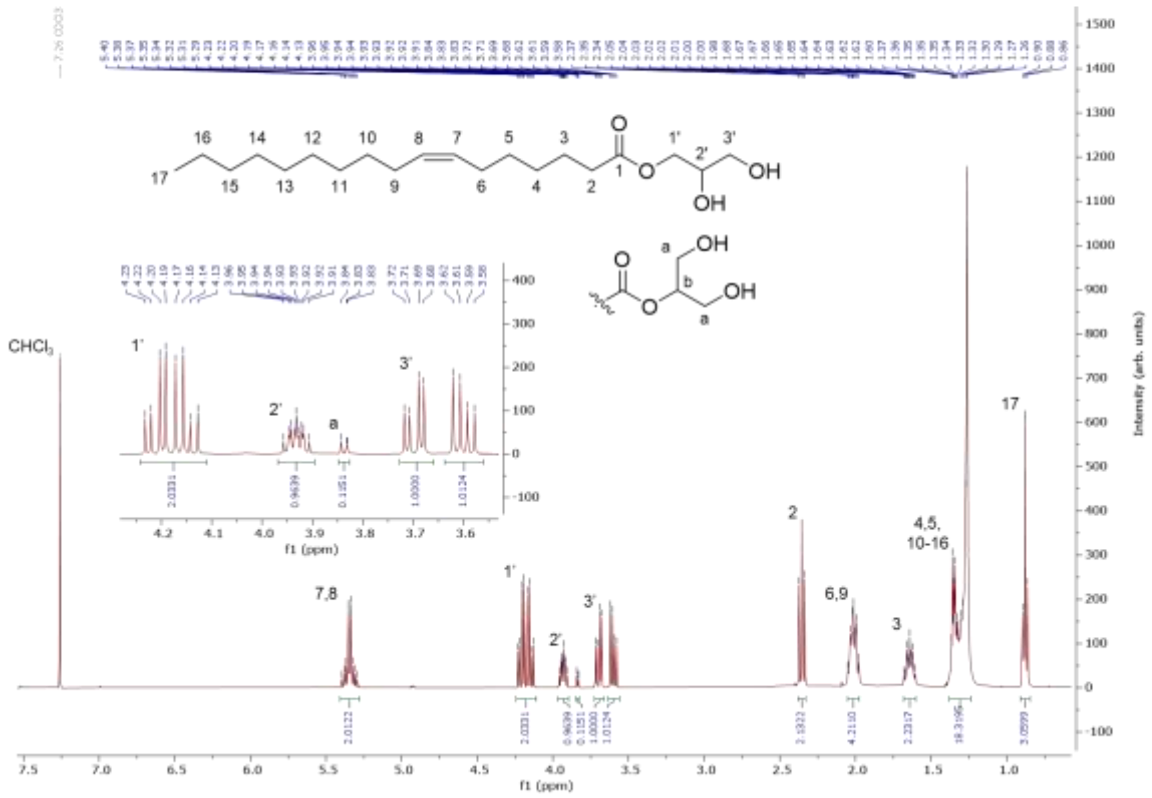

Figure S3. ^{1}H NMR of synthetic 7.10 MAG and estimation of 2-MAG content. Data was collected on a Bruker Avance 400 (400.13 MHz) with a sample of 5 mg synthetic 7.10 MAG in 600 µL of deuterated chloroform ($CDCl_3$). The sample contains both the 1-MAG and 2-MAG isomers, the structure or partial structure of which are included in the figure. The inset shows an expanded view of the glycerol region, which contains tell-tale resonances associated with the 2-MAG. Protons are labelled by numbers, with letters indicating the 2-MAG resonances. The spectrum has a signal-to-noise ratio of >2,000. This, coupled with the lack of unexplained resonances, indicates that the sample is of very high purity. The lipid had a pale-yellow color that cannot be accounted for by additional resonances in the NMR spectrum. The mol% of 2-MAG in the mixture of isomers is estimated by NMR. The doublet of resonances at ~3.85 ppm arising from 4 methylene protons in the 2-MAG isomer (indicated by *a* in the 2-MAG partial structure) integrates to 0.1151 (blue numbers on the x-axis at ~3.85 ppm). This value has been scaled relative to the integrated value of the doublet of doublet resonance centered at ~3.7 ppm arising from one of the diastereotopic protons at the *sn*-3 carbon of the 1-MAG glycerol unit (indicated by 3' in the 1-MAG structure), which has been normalized to an integrated value of 1.0000 (blue numbers at 3.7 ppm). This means that the signal at 3.85 ppm for the 2-MAG isomer representing 4 protons is 11.51% of the signal for the 1 proton in the 1-MAG isomer at 3.7 ppm. Therefore, the 2- MAG concentration as a mol% of the 1- isomer content in the sample is (11.51/4 =) 2.88 mol%. The 2-MAG content in the sample as a whole is (2.88 (2-MAG)/[102.88 (1-MAG + 2-MAG)] * 100 =) 2.80 mol%.

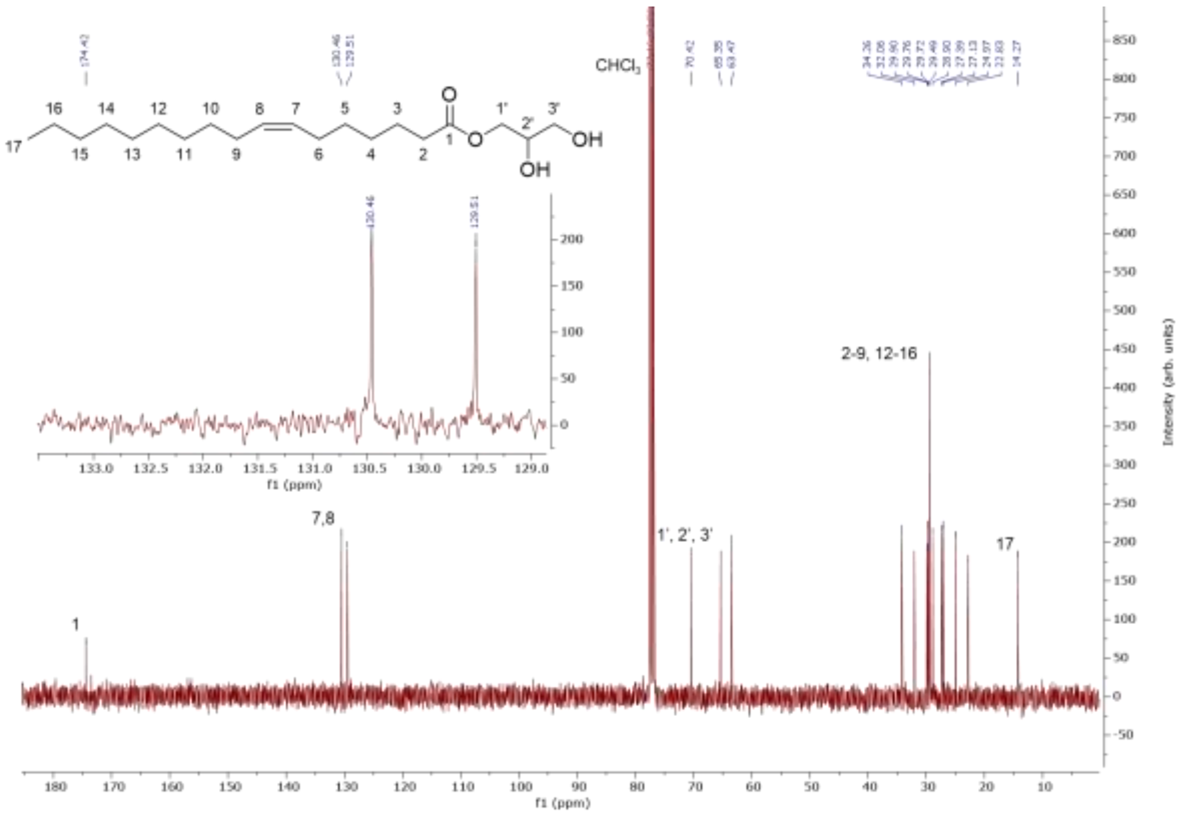

Figure S4. ^{13}C NMR spectrum of synthetic 7.10 MAG. Data was collected on a Bruker Avance 400 (400.13 MHz) with a sample of 5 mg synthetic 7.10 MAG in 600 µL deuterated chloroform ($CDCl_3$). The lipid was synthesized *via* a Suzuki-Miyaura cross coupling, designed to give only the *cis*-alkene. The inset shows an expanded view of the alkene region. The *trans*-alkene (here not distinguishable from noise) would appear at approximately 130.6 and 130.9 ppm. (Coleman *et al.* 2004) The *trans*-alkene content is estimated at $\leq$1.09 mol% based on a signal-to-noise ratio of 91.95 calculated using MestReNova processing software.

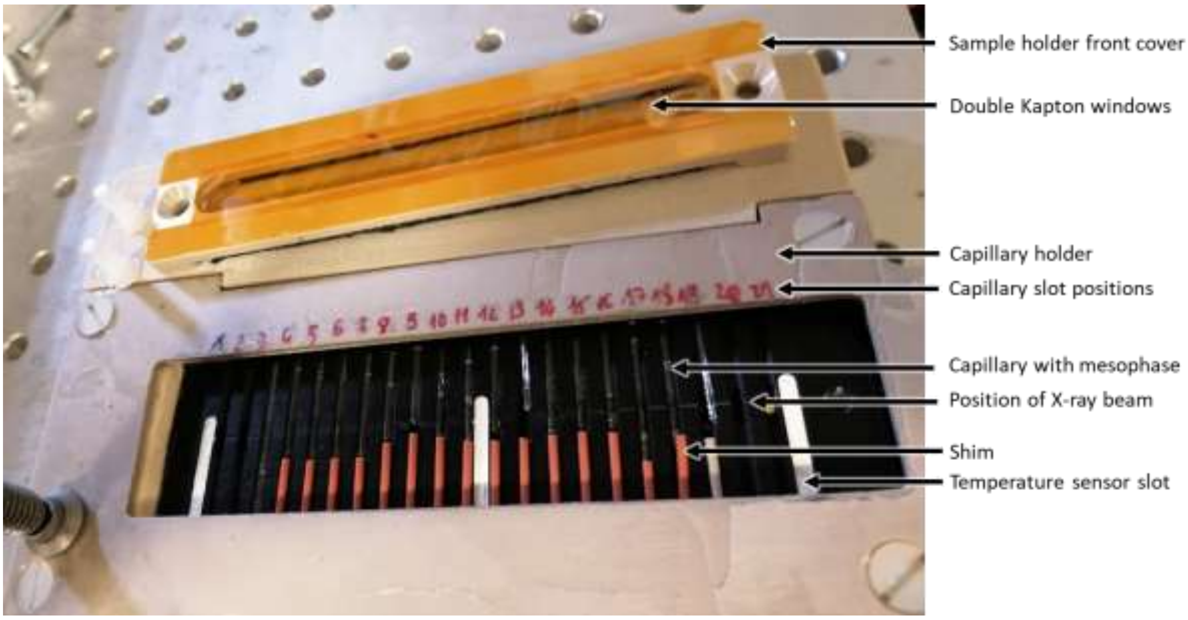

Figure S5. Dismantled Static Capillary Holder. The holder accommodates 21 sample capillaries (holder slots 1-21 spaced 5 mm apart) and 3 temperature sensors (A-C). Sensors A and C are next to holder slots 1 and 21, respectively. Sensor B is between slots 11 and 12. Shims are used to position mesophase in capillaries in the X-ray beam. The holder has front and back covers each with double Kapton windows. The sample facing surface of the front cover is coated with sponge that holds the shims and capillaries in position during data collection.

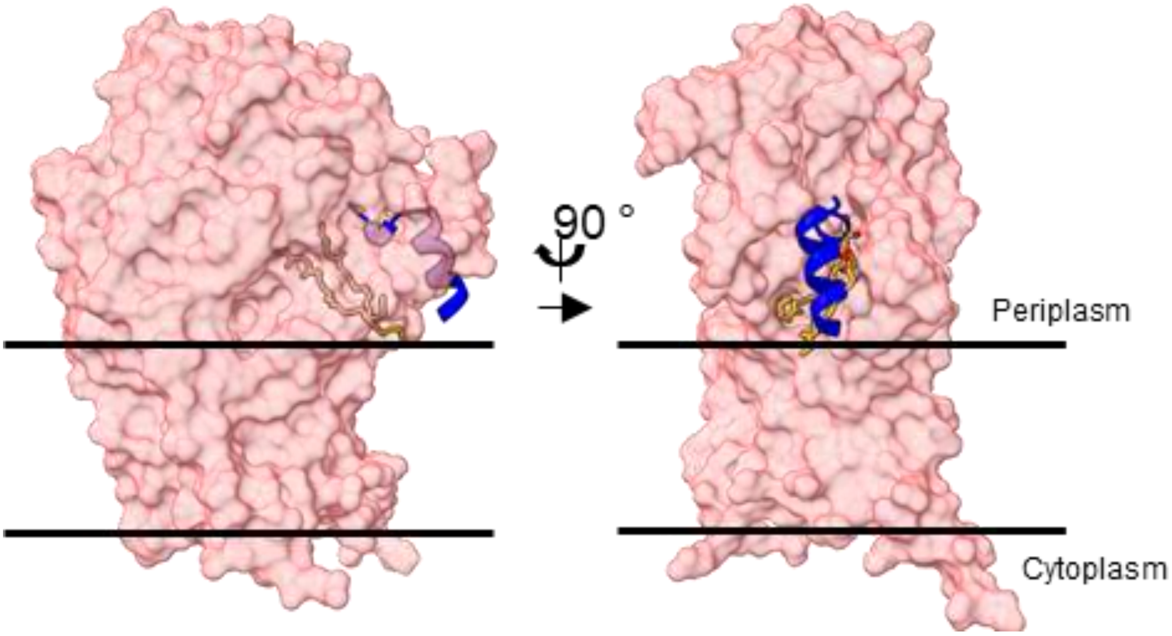

Figure S6. Novel crystal packing in the Lnt 7.10 MAG structure may mimic substrate engagement. Lnt is shown in transparent surface representation (pink). Two 7.10 MAG molecules are seen in the binding pocket (yellow carbons, red oxygens with a black outline). The first 15 N-terminal residues of a neighbouring Lnt molecule is shown in blue cartoon representation with a black outline, extending into the binding pocket

SMovie 1. Step-by-step procedures for SAXS/WAXS sample preparation and X-ray capillary loading and sealing are covered in this movie.
